# Supplementary material for: The Effect of Prebiotics, Alone or as Part of Synbiotics, on Cardiometabolic Parameters in Women with Polycystic Ovary Syndrome: A Systematic Review and Meta-Analysis of Randomized Controlled Trials
Source: Biomedicines. 2025 Jan 13;13(1):177. doi: 10.3390/biomedicines13010177 (PMC11760460; doi:10.3390/biomedicines13010177)
Supplement: Supplementary file 1 [file biomedicines-13-00177-s001.zip › Table S1_Search strategies.pdf]

The effect of prebiotics, alone or as part of synbiotics, on cardiometabolic parameters in women with polycystic ovary syndrome: a systematic review and meta-analysis of randomized controlled trials

Elham Razmpoosh [1*], Mala S. Sivanandy [2*], Alan M. Ehrlich [3] •
[1] Department of Health Research Methods, Evidence and Impact (HEI), McMaster University, Hamilton, Canada.
[2] PCOS Center, Division of Endocrinology, Beth Israel Deaconess Medical Center, Harvard Medical School, Boston, USA.
[3] Department of Family Medicine and Community Health, UMass Chan Medical School, Worcester, MA and EBSCO Information Services, Ipswich MA, USA.

- **Dr. Alan M. Ehrlich, MD, FAAFP**
  Department of Family Medicine and Community Health, UMass Chan Medical School, Worcester MA, and EBSCO Information Services, USA
  Tel: +1-508-439-1157
  Email: aehrlich@ebsco.com
  Orchid ID: 0009-0002-6052-9902

[*] Elham Razmpoosh and Mala S. Sivanandy contributed equally to this work.

Supplementary Table S1 Search strategies

**PubMed**

(Fibre*[tiab] OR fiber[tiab] OR fibrous[tiab] OR prebiotic*[tiab] OR inulin[tiab] OR resistant starch[tiab] OR resistant-starch[tiab] OR pectin*[tiab] OR cellulose*[tiab] OR lignin[tiab] OR gum[tiab] OR plant mucilage[tiab] OR plant-mucilage[tiab] OR roughage[tiab] OR hemicellulose*[tiab] OR psyllium[tiab] OR beta-glucan*[tiab] OR beta glucan [tiab] OR whole grain*[tiab] OR bran[tiab] OR crude-fibre[tiab] OR soluble-fibre[tiab] OR soluble fibre*[tiab] OR fermentable-fibre[tiab] OR fermentable-fibre[tiab] OR insoluble-fibre[tiab] OR dextrin*[tiab] OR oligosaccharide*[tiab] OR wheat-bran[tiab] OR polysaccharide*[tiab] OR non-starch polysaccharide*[tiab] OR arabinoxylan*[tiab] OR fructan*[tiab] OR polyuronide*[tiab] OR raffinose[tiab] OR polydextrose[tiab] OR xantham gum*[tiab] OR xantham-gum*[tiab] OR guar gum*[tiab] OR guar-gum*[tiab] OR plant gum*[tiab] OR plant-gum*[tiab] OR methylcellulose*[tiab] OR arabinogalactan*[tiab] OR galactomannan*[tiab] OR synbiotic*[tiab] OR symbiotic*[tiab] OR Microbiota*[Tiab] OR Microbiome[Tiab] OR Microbiome*[Tiab] OR Gut microflora*[Tiab] OR Gut-microflora*[Tiab] OR Gut-microbiota*[Tiab] OR Human-Microbiome*[Tiab] OR cultured milk products[MeSH Terms] OR milk product[Title/Abstract] OR yogurt*[Title/Abstract] OR kefir*[Title/Abstract] OR dairy product*[Title/Abstract] OR Fermented Foods and Beverages[MeSH Terms] OR Fermented milk[Title/Abstract] OR Nissle[Title/Abstract] OR Fermented Foods[Title/Abstract] OR buttermilk[Title/Abstract] OR lassi[Title/Abstract] OR doogh[Title/Abstract] OR dough[Title/Abstract] OR dahi[Title/Abstract] OR amasi[Title/Abstract] OR filmjolk[Title/Abstract] OR chal[Title/Abstract] OR yeast*[Title/Abstract]) OR synbiotics [MeSH Terms] OR cultured milk products [MeSH Terms] OR Dietary Fiber [MeSH terms] OR prebiotics[MeSH Terms] OR "Dietary Approaches To Stop Hypertension"[Mesh] OR DASH [tiab] OR Dietary Approaches to Stop Hypertension [tiab] OR mediterranean [tiab] OR MED diet* [tiab] OR MD diet* [tiab] OR "Diet, Mediterranean"[Mesh]) AND (Polycystic Ovary Syndrome[MeSH] OR Polycystic Ovary Syndrome*[tiab] OR Ovary Syndrome, Polycystic[Tiab] OR Syndrome, Polycystic Ovary[Tiab] OR Stein-Leventhal Syndrome[Tiab] OR Stein Leventhal Syndrome [Tiab] OR Syndrome, Stein-Leventhal[Tiab] OR Sclerocystic Ovarian Degeneration[Tiab] OR Ovarian Degeneration, Sclerocystic [Tiab] OR Sclerocystic Ovary Syndrome [Tiab] OR Polycystic Ovarian Syndrome*[Tiab] OR Ovarian Syndrome, Polycystic[Tiab] OR Sclerocystic Ovar* [Tiab] OR Ovary, Sclerocystic[Tiab] OR Sclerocystic Ovary[Tiab])
**Scopus**

(TITLE-ABS-KEY (fibre*) OR TITLE-ABS-KEY (fiber*) OR TITLE-ABS-KEY (fibrous) OR TITLE-ABS-KEY (prebiotic*) OR TITLE-ABS-KEY (inulin) OR TITLE-ABS-KEY ("resistant starch") OR TITLE-ABS-KEY (pectin*) OR TITLE-ABS-KEY (cellulose*) OR TITLE-ABS-KEY (lignin) OR TITLE-ABS-KEY (gum) OR TITLE-ABS-KEY ("plant mucilage") OR TITLE-ABS-KEY (roughage) OR TITLE-ABS-KEY (hemicellulose*) OR TITLE-ABS-KEY (psyllium) OR TITLE-ABS-KEY ("beta-glucans") OR TITLE-ABS-KEY ("whole grain*") OR TITLE-ABS-KEY (bran) OR TITLE-ABS-KEY (dextrin*) OR TITLE-ABS-KEY (oligosaccharide*) OR TITLE-ABS-KEY ("wheat bran") OR TITLE-ABS-KEY (polysaccharide*) OR TITLE-ABS-KEY ("non-starch polysaccharide*") OR TITLE-ABS-KEY (arabinoxylan) OR TITLE-ABS-KEY (fructan*) OR TITLE-ABS-KEY (polyuronide) OR TITLE-ABS-KEY (raffinose) OR TITLE-ABS-KEY (polydextrose) OR TITLE-ABS-KEY ("xantham gum") OR TITLE-ABS-KEY ("guar gum") OR TITLE-ABS-KEY ("plant gum*") OR TITLE-ABS-KEY (methylcellulose) OR TITLE-ABS-KEY (arabinogalactan) OR TITLE-ABS-KEY (galactomannan) OR TITLE-ABS-KEY (synbiotic*) OR TITLE-ABS-KEY (symbiotic*) OR TITLE-ABS-KEY (Microbiota*) OR TITLE-ABS-KEY (Microbiome*) OR TITLE-ABS-KEY (“Gut microflora*”) OR TITLE-ABS-KEY (“Gut-microflora*”) OR TITLE-ABS-KEY (“Gut-microbiota*”) OR TITLE-ABS-KEY (Human-Microbiome*) OR TITLE-ABS-KEY (“milk product*”) OR TITLE-ABS-KEY (yogurt*) OR TITLE-ABS-KEY (kefir*) OR TITLE-ABS-KEY (“dairy product*”) OR (TITLE-ABS-KEY (dash) OR TITLE-ABS-KEY ("Dietary Approaches to Stop Hypertension") OR (TITLE-ABS-KEY (mediterranean) OR TITLE-ABS-KEY ("MED diet*") OR TITLE-ABS-KEY ("MD diet*")) AND (TITLE-ABS-KEY (“Polycystic Ovary Syndrome*”) OR TITLE-ABS-KEY (“Ovary Syndrome, Polycystic”) OR TITLE-ABS-KEY (“Syndrome, Polycystic Ovary”) OR TITLE-ABS-KEY (“Stein-Leventhal Syndrome*”) OR TITLE-ABS-KEY (“Stein Leventhal Syndrome”) OR TITLE-ABS-KEY (“Syndrome, Stein-Leventhal”) OR TITLE-ABS-KEY (“Sclerocystic Ovarian Degeneration”) OR TITLE-ABS-KEY (“Ovarian Degeneration, Sclerocystic”) OR TITLE-ABS-KEY (“Sclerocystic Ovary Syndrome”) OR TITLE-ABS-KEY (“Polycystic Ovarian Syndrome*”) OR TITLE-ABS-KEY (“Ovarian Syndrome, Polycystic”) OR TITLE-ABS-KEY (“Sclerocystic Ovar*”) OR TITLE-ABS-KEY (“Ovary, Sclerocystic”) OR TITLE-ABS-KEY (“Sclerocystic Ovary”))
**ISI Web of Science**
ALL=(Fibre)) OR ALL=(Fiber)) OR ALL=(Fibrous) OR ALL=(prebiotic*) OR ALL=(Inulin) OR ALL=("resistant starch") OR ALL=(pectin*) OR ALL=(cellulose*) OR ALL=(lignin) OR ALL=(gum) OR ALL=("Plant Mucilage") OR ALL=(roughage) OR ALL=(Hemicellulose*) OR ALL=(psyllium) OR ALL=(beta-Glucans) OR ALL=("Whole grain*") OR ALL=(Bran)) OR ALL=(Hemicellulose) OR ALL=("Crude fibre") OR ALL=("soluble fibre") OR ALL=("fermentable fibre") OR ALL=("insoluble fibre") OR ALL=(Dextrin*) OR ALL=(oligosaccharide*) OR ALL=("wheat bran") OR ALL=(polysaccharide*) OR ALL=("non-starch polysaccharide*") OR ALL=(arabinoxylan) OR ALL=(fructan*) OR ALL=(polyuronide) OR ALL=(raffinose) OR ALL=(polydextrose) OR ALL=("xanthan gum") OR ALL=("guar gum") OR ALL=("Plant gum*") OR ALL=(synbiotic*) OR ALL=(symbiotic*) OR ALL=(Microbiota*) OR ALL=(Microbiome) OR ALL=(Microbiome*) OR ALL=(“Gut microflora*”) OR ALL=(“Gut-microflora*") OR ALL=(“Gut-microbiota*”) OR ALL=(“Human-Microbiome*”) OR ALL=(“cultured milk product*”) OR ALL=(“milk product*”) OR ALL=(yogurt*) OR ALL=(kefir*) OR ALL=(“dairy product*”) OR

ALL=(“Fermented Foods and Beverage*”) OR ALL=(“Fermented milk*”) OR ALL=(Nissle) OR ALL=(“Fermented Food*”) OR ALL=(buttermilk*) OR ALL=(lassi) OR ALL=(doogh) OR ALL=(dough) OR ALL=(dahi) OR ALL=(amasi) OR ALL=(filmjolk) OR ALL=(chal) OR ALL=(yeast*) OR (ALL=(methylcellulose) OR ALL=(arabinogalactan) OR ALL=(galactomannan) OR **ALL=(mediterranean) OR ALL=("MED diet*") OR ALL=("MD diet*")** OR All=(dash) OR All=("Dietary Approaches to Stop Hypertension") AND ALL=(“Polycystic Ovary Syndrome*”) OR ALL=(“Ovary Syndrome, Polycystic”) OR ALL=(“Syndrome, Polycystic Ovary”) OR ALL=(“Stein-Leventhal Syndrome*”) OR ALL=(“Stein Leventhal Syndrome”) OR ALL=(“Syndrome, Stein-Leventhal”) OR ALL=(“Sclerocystic Ovarian Degeneration”) OR ALL=(“Ovarian Degeneration, Sclerocystic”) OR ALL=(“Sclerocystic Ovary Syndrome”) OR ALL=(“Polycystic Ovarian Syndrome*”) OR ALL=(“Ovarian Syndrome, Polycystic”) OR ALL=(“Sclerocystic Ovar*”) OR ALL=(“Ovary, Sclerocystic”) OR ALL=(“Sclerocystic Ovary”)

Embase

2	Fiber.mp.
2	Fibre.mp.
3	Fibrous.mp.
4	prebiotic*.mp.
5	inulin/ or Inulin.mp.
6	resistant starch.mp.
7	pectin/ or pectin*.mp.
8	cellulose*.mp. or cellulose/
9	lignin/ or lignin.mp.
10	Gum.mp.
11	Plant Mucilage.mp. or mucilage/
12	roughage.mp. or roughage/
13	hemicellulose/ or Hemicellulose*.mp.
14	psyllium.mp. or ispagula/
15	beta-Glucans.mp. or beta glucan/
16	Whole grain*.mp. or whole grain/
17	bran/ or Bran.mp.
18	hemicellulose/ or Hemicellulose.mp.
19	Crude fibre.mp.
20	soluble fibre.mp.
21	fermentable fibre.mp.
22	insoluble fibre.mp.
23	dextrin/ or Dextrin*.mp.
24	oligosaccharide/ or oligosaccharide*.mp.
25	wheat bran.mp. or wheat bran/
26	polysaccharide/ or polysaccharide*.mp.
27	non-starch polysaccharide*.mp.
28	arabinoxylan.mp. or arabinoxylan/
29	fructan/ or fructan*.mp.
30	Polyuronide.mp.
31	raffinose.mp. or raffinose/
32	Polydextrose.mp.

33	xanthan gum.mp.
34	guar gum.mp. or guar gum/
35	plant gum/ or Plant gum*.mp.
36	methylcellulose.mp. or methylcellulose/
37	arabinogalactan.mp. or arabinogalactan/
38	galactomannan.mp. or galactomannan/
39	synbiotic*.mp.
40	symbiotic*.mp.
41	Gut microflora*.mp.
42	microbiota.mp.
43	microbiome*.mp.
44	Human-Microbiome*.mp.
45	cultured milk product*.mp.
46	milk product*.mp.
47	dairy product*.mp.
48	kefir*.mp.
49	yogurt*.mp.
50	"Fermented food*".mp.
51	"Fermented Foods and Beverage*".mp.
52	Nissle*.mp.
53	buttermilk*.mp.
54	doogh*.mp.
55	dough*.mp.
56	lassi*.mp.
57	dahi*.mp.
58	amasi*.mp.
59	chal.mp.
60	yeast*.mp.
61	Polycystic Ovary Syndrome*.mp.
62	Ovary Syndrome, Polycystic.mp.
63	Syndrome, Polycystic Ovary.mp.
64	Polycystic Ovarian Syndrome*.mp.
65	Stein-Leventhal Syndrome*.mp.
66	Stein Leventhal Syndrome*.mp.
67	Syndrome, Stein-Leventhal.mp.
68	Sclerocystic Ovarian Degeneration.mp.
69	Ovarian Degeneration, Sclerocystic.mp.
70	Sclerocystic Ovary Syndrome.mp.
71	Ovarian Syndrome, Polycystic.mp.
72	Ovary, Sclerocystic.mp.
73	Sclerocystic Ovary.mp.
74	1 or 2 or 3 or 4 or 5 or 6 or 7 or 8 or 9 or 10 or 11 or 12 or 13 or 14 or 15 or 16 or 17 or 18 or 19 or 20 or 21 or 22 or 23 or 24 or 25 or 26 or 27 or 28 or 29 or 30 or 31 or 32 or 33 or 34 or 35 or 36 or 37 or 38 or 39 or 40 or 41 or 42 or 43 or 44 or 45 or 46 or 47 or 48 or 49 or 50 or 51 or 52 or 53 or 54 or 55 or 56 or 57 or 58 or 59 or 60
75	61 or 62 or 63 or 64 or 65 or 66 or 67 or 68 or 69 or 70 or 71 or 72 or 73

76	74 and 75

Cochrane Central Register of Controlled Trials

Prebiotics/synbiotics

#1

(fibre*):ti,ab,kw OR (fiber*):ti,ab,kw OR (fibrous):ti,ab,kw OR (prebiotic*):ti,ab,kw OR (inulin):ti,ab,kw OR ("resistant starch"):ti,ab,kw OR (pectin*):ti,ab,kw OR (cellulose*):ti,ab,kw OR (lignin):ti,ab,kw OR (gum):ti,ab,kw OR ("plant mucilage"):ti,ab,kw OR (roughage):ti,ab,kw OR (hemicellulose*):ti,ab,kw OR (psyllium):ti,ab,kw OR ("beta-glucans"):ti,ab,kw OR ("whole grain*"):ti,ab,kw OR (bran):ti,ab,kw OR (dextrin*):ti,ab,kw OR (oligosaccharide*):ti,ab,kw OR ("wheat bran"):ti,ab,kw OR (polysaccharide*):ti,ab,kw OR ("non-starch polysaccharide*"):ti,ab,kw OR (arabinoxylan):ti,ab,kw OR (fructan*):ti,ab,kw OR (polyuronide):ti,ab,kw OR (raffinose):ti,ab,kw OR (polydextrose):ti,ab,kw OR ("xantham gum"):ti,ab,kw OR ("guar gum"):ti,ab,kw OR ("plant gum*"):ti,ab,kw OR (methylcellulose):ti,ab,kw OR (arabinogalactan):ti,ab,kw OR (galactomannan):ti,ab,kw OR (synbiotic*):ti,ab,kw OR (symbiotic*):ti,ab,kw OR (Microbiota*):ti,ab,kw OR (Microbiome*):ti,ab,kw OR ("Gut microflora*"):ti,ab,kw OR ("Gut-microflora*"):ti,ab,kw OR ("Gut-microbiota*"):ti,ab,kw OR (Human-Microbiome*):ti,ab,kw OR ("milk product*"):ti,ab,kw OR (yogurt*):ti,ab,kw OR (kefir*):ti,ab,kw OR ("dairy product*"):ti,ab,kw OR (DASH):ti,ab,kw OR (Dietary Approaches To Stop Hypertension):ti,ab,kw OR (Mediterranean):ti,ab,kw OR ("MED diet"):ti,ab,kw OR ("MD diet*):ti,ab,kw

#2

MeSH descriptor: [Synbiotics] explode all trees

#3

MeSH descriptor: [Cultured Milk Products] explode all trees

#4

MeSH descriptor: [Dietary Fiber] explode all trees

#5

MeSH descriptor: [Prebiotics] explode all trees

6#

MeSH descriptor: [Dietary Approaches To Stop Hypertension] explode all trees

7#

MeSH descriptor: [Diet, Mediterranean] explode all trees

#8

#1 OR #2 OR #3 OR #4 OR #5 OR #6 OR #7 OR #8

#9

("Polycystic Ovary Syndrome*"):ti,ab,kw OR ("Ovary Syndrome, Polycystic"):ti,ab,kw OR ("Syndrome, Polycystic Ovary"):ti,ab,kw OR ("Stein-Leventhal Syndrome*"):ti,ab,kw OR ("Stein Leventhal Syndrome"):ti,ab,kw OR ("Syndrome, Stein-Leventhal"):ti,ab,kw OR ("Sclerocystic Ovarian Degeneration"):ti,ab,kw OR ("Ovarian Degeneration, Sclerocystic"):ti,ab,kw OR ("Sclerocystic Ovary Syndrome"):ti,ab,kw OR ("Polycystic Ovarian Syndrome*"):ti,ab,kw OR ("Ovarian Syndrome, Polycystic"):ti,ab,kw OR ("Sclerocystic Ovar*"):ti,ab,kw OR ("Ovary, Sclerocystic"):ti,ab,kw OR ("Sclerocystic Ovary"):ti,ab,kw

#10

MeSH descriptor: [Polycystic Ovary Syndrome] explode all trees

#11
#9 OR #10
#12
#8 AND #11
