# Supplementary material for: The Effect of Prebiotics, Alone or as Part of Synbiotics, on Cardiometabolic Parameters in Women with Polycystic Ovary Syndrome: A Systematic Review and Meta-Analysis of Randomized Controlled Trials
Source: Biomedicines. 2025 Jan 13;13(1):177. doi: 10.3390/biomedicines13010177 (PMC11760460; doi:10.3390/biomedicines13010177)
Supplement: Supplementary file 1 [file biomedicines-13-00177-s001.zip › Table S2_RoB 2 Quality assessment.pdf]

The effect of prebiotics, alone or as part of synbiotics, on cardiometabolic parameters in women with polycystic ovary syndrome: a systematic review and meta-analysis of randomized controlled trials

Elham Razmpoosh [1*], Mala S. Sivanandy [2*], Alan M. Ehrlich [3] •
[1] Department of Health Research Methods, Evidence and Impact (HEI), McMaster University, Hamilton, Canada.
[2] PCOS Center, Division of Endocrinology, Beth Israel Deaconess Medical Center, Harvard Medical School, Boston, USA.
[3] Department of Family Medicine and Community Health, UMass Chan Medical School, Worcester, MA and EBSCO Information Services, Ipswich MA, USA.

- **Dr. Alan M. Ehrlich, MD, FAAFP**
  Department of Family Medicine and Community Health, UMass Chan Medical School, Worcester MA, and EBSCO Information Services, USA
  Tel: +1-508-439-1157
  Email: aehrlich@ebsco.com
  Orchid ID: 0009-0002-6052-9902

[*] Elham Razmpoosh and Mala S. Sivanandy contributed equally to this work.

Supplementary Table S2 Study quality and risk of bias assessment using the Cochrane collaboration tool (RoB 2)

Author (Year)	Randomization Process	Deviations from the intended intervention		Missing outcome data	Measurement of the outcomes	Selection of the reported results	Overall quality [b]
		Effect of assignment to intervention	Effect of adhering to intervention				
Asemi et al. (2014)	–	✓	✓	✓	✓	✓	–
Asemi et al. (2015)	–	✓	✓	✓	✓	✓	–
Azadi et al. (2017)	–	–	✓	✓	–	✓	✗
Chudzika et al. (2021)	–	✓	✓	✓	✓	✓	–
Darvishi et al. (2021)	–	✓	✓	✓	✓	✓	–

Duo et al. (2023)	–	–	✓	✓	–	✓	✗
Esmaeilinezhad et al. (2019)	✓	–	✓	✓	✓	✓	–
Esmaeilinezhad et al. (2020)	✓	✓	✓	✓	✓	✓	✓
Foroozanfard et al. (2017)	✓	✓	✓	✓	✓	–	–
Gholizadeh Shamasbi et al. (2019)	✓	✓	✓	✓	✓	✓	✓
Gholizadeh Shamasbi et al. (2018)	✓	✓	✓	✓	✓	✓	✓
Hariri et al. (2023)	✓	✓	✓	✓	✓	✓	✓
Hariri et al. (2024)	✓	✓	✓	✓	✓	✓	✓
Karimi et al. (2020)	✓	✓	✓	✓	✓	✓	✓
Karimi et al. (2018)	✓	✓	✓	✓	✓	✓	✓

Nasri et al. (2018)	✓	✓	✓	✓	✓	✓	✓
Pourbehi et al. (2018)	✓	✗	✓	✓	–	✓	✗
Samimi et al. (2019)	✓	✓	✓	✓	✓	✓	✓
Ziaei et al. (2022)	✓	✓	✓	✓	✓	✓	✓
Ziaei et al. (2024)	✓	✓	✓	✓	✓	✓	✓
