# Supplementary material for: The Effect of Prebiotics, Alone or as Part of Synbiotics, on Cardiometabolic Parameters in Women with Polycystic Ovary Syndrome: A Systematic Review and Meta-Analysis of Randomized Controlled Trials
Source: Biomedicines. 2025 Jan 13;13(1):177. doi: 10.3390/biomedicines13010177 (PMC11760460; doi:10.3390/biomedicines13010177)
Supplement: Supplementary file 1 [file biomedicines-13-00177-s001.zip › Table S3_GRADE.pdf]

Synbiotics/Probiotics compared to Control in women with PCOS

Bibliography:

Certainty assessment							Summary of findings				
**Participants (studies) Follow-up**	**Risk of bias**	**Inconsistency**	**Indirectness**	**Imprecision**	**Publication bias**	**Overall certainty of evidence**	**Study event rates (%)**		**Relative effect (95% CI)**	**Anticipated absolute effects**	
							**With Control**	**With Synbiotics/Probiotics**		**Risk with Control**	**Risk difference with Synbiotics/Probiotics**
**Body Mass Index (follow-up: range 8 weeks to 12 weeks; assessed with: Formula)**											
853 (14 RCTs)	serious[a]	not serious	not serious	not serious	strong association	⊕⊕⊕⊕ High[a]	398	455	-	398	MD **0.532 Kg/m2 lower** (0.695 lower to 0.369 lower)
**Weight (follow-up: range 8 weeks to 12 weeks; assessed with: A standard scale)**											
853 (14 RCTs)	serious[a]	not serious	not serious	not serious	publication bias strongly suspected strong association[b]	⊕⊕⊕◯ Moderate[a,b]	398	455	-	398	MD **2.383 Kg lower** (2.818 lower to 1.949 lower)
**Waist circumference (follow-up: range 8 weeks to 12 weeks; assessed with: a standard scale)**											
463 (8 RCTs)	serious[a]	serious[c]	not serious	not serious	publication bias strongly suspected[b]	⊕◯◯◯ Very low[a,b,c]	230	233	-	230	MD **3.461 cm lower** (4.613 lower to 2.309 lower)
**Hip circumference (follow-up: range 8 weeks to 12 weeks; assessed with: a standard scale)**											
565 (9 RCTs)	serious[a]	serious[c]	not serious	serious[d]	none	⊕◯◯◯ Very low[a,c,d]	279	286	-	279	MD **1.203 Cm lower** (2.992 lower to 0.586 higher)
**Wais-to-hip ratio (follow-up: range 8 weeks to 12 weeks; assessed with: Formula)**											

Synbiotics/Probiotics compared to Control in women with PCOS
Bibliography:

Certainty assessment							Summary of findings				
401 (7 RCTs)	serious[a]	not serious	not serious	not serious	none	⨁⨁⨁◯ Moderate[a]	199	202	-	199	MD **0.018 lower** (0.03 lower to 0.005 lower)

Fat Mass (follow-up: range 8 weeks to 12 weeks; assessed with: a standard scale)

202 (4 RCTs)	serious[a]	serious[c]	not serious	not serious	none	⨁⨁◯◯ Low[a,c]	100	102	-	100	MD **2.538 Kg lower** (4.403 lower to 0.673 lower)

High density lipoprotein cholesterol (follow-up: range 8 weeks to 12 weeks; assessed with: Laboratory test)

621 (10 RCTs)	serious[a]	serious[c]	not serious	serious[d]	publication bias strongly suspected[b]	⨁◯◯◯ Very low[a,b,c,d]	305	316	-	305	MD **2.101 mg/dL higher** (0.224 lower to 4.426 higher)

Low density lipoprotein cholesterol (follow-up: range 8 weeks to 12 weeks; assessed with: Laboratory test)

621 (10 RCTs)	serious[a]	serious[c]	not serious	not serious	none	⨁⨁◯◯ Low[a,c]	305	316	-	305	MD **11.228 mg/dL lower** (19.356 lower to 3.1 lower)

Total cholesterol (follow-up: range 8 weeks to 12 weeks; assessed with: Laboratory test)

621 (10 RCTs)	serious[a]	serious[c]	not serious	not serious	none	⨁⨁◯◯ Low[a,c]	305	316	-	305	MD **8.504 mg/dL lower** (14.434 lower to 2.574 lower)

Triglycerides (follow-up: range 8 weeks to 12 weeks; assessed with: Laboratory test)

Synbiotics/Probiotics compared to Control in women with PCOS

Bibliography:

Certainty assessment							Summary of findings				
621 (10 RCTs)	serious [a]	not serious	not serious	not serious	none	⊕⊕⊕◯ Moderate[a]	305	316	-	305	MD **11.601 mg/dL lower** (18.252 lower to 4.951 lower)

Fasting plasma glucose (follow-up: range 8 weeks to 12 weeks; assessed with: Laboratory test)

686 (11 RCTs)	serious [a]	serious[c]	not serious	not serious	none	⊕⊕◯◯ Low[a,c]	342	344	-	342	MD **4.142 mg/dL lower** (7.233 lower to 1.051 lower)

Fasting inulin (follow-up: range 8 weeks to 12 weeks; assessed with: Laboratory test)

624 (10 RCTs)	serious [a]	serious[c]	not serious	not serious	none	⊕⊕◯◯ Low[a,c]	311	313	-	311	MD **2.804 mg/dL lower** (4.33 lower to 1.278 lower)

Quantitative Insulin Sensitivity Check Index (follow-up: range 8 weeks to 12 weeks; assessed with: Formula)

457 (7 RCTs)	serious [a]	serious[c]	not serious	not serious	none	⊕⊕◯◯ Low[a,c]	228	229	-	228	MD **0.018 higher** (0.006 higher to 0.029 higher)

Homeostasis Model Assessment for Insulin Resistance (follow-up: range 8 weeks to 12 weeks; assessed with: Formula)

615 (9 RCTs)	serious [a]	serious[c]	not serious	serious[d]	none	⊕◯◯◯ Very low[a,c,d]	292	323	-	292	MD **0.376 lower** (0.993 lower to 0.24 higher)

Total testosterone (follow-up: range 8 weeks to 12 weeks; assessed with: Laboratory test)

Synbiotics/Probiotics compared to Control in women with PCOS

Bibliography:

Certainty assessment							Summary of findings				
416 (7 RCTs)	serious [a]	serious[c]	not serious	not serious	none	⊕⊕◯◯ Low[a,c]	207	209	-	207	MD **0.193 IU/L lower** (0.381 lower to 0.006 lower)
**Follicle stimulating hormone (follow-up: range 9 weeks to 12 weeks; assessed with: Laboratory test)**											
191 (4 RCTs)	serious [a]	not serious	not serious	not serious	publication bias strongly suspected[b]	⊕⊕◯◯ Low[a,b]	95	96	-	95	MD **1.02 IU/L higher** (0.475 higher to 1.564 higher)
**luteinizing hormone (follow-up: range 8 weeks to 12 weeks; assessed with: Laboratory test)**											
191 (4 RCTs)	serious [a]	serious[c]	not serious	serious[d]	none	⊕◯◯◯ Very low[a,c,d]	95	96	-	95	MD **1.05 IU/L higher** (0.392 lower to 2.493 higher)
**Sex Hormone-Binding Globulin (follow-up: range 8 weeks to 12 weeks; assessed with: Laboratory test)**											
314 (6 RCTs)	serious [a]	very serious[c]	not serious	very serious[d]	none	⊕◯◯◯ Very low[a,c,d]	161	153	-	161	MD **14.329 IU/L higher** (5.266 lower to 33.924 higher)
**Dehydroepiandrosterone sulfate (follow-up: range 8 weeks to 12 weeks; assessed with: Laboratory test)**											
161 (3 RCTs)	serious [a]	serious[c]	not serious	serious[d]	none	⊕◯◯◯ Very low[a,c,d]	80	81	-	80	MD **0.179 µmol/L higher** (0.381 lower to 0.739 higher)
**Free androgen index (follow-up: range 8 weeks to 12 weeks; assessed with: Laboratory test)**											
285 (4 RCTs)	serious [a]	serious[c]	not serious	not serious	none	⊕⊕◯◯ Low[a,c]	142	143	-	142	MD **0.206 IU/L lower** (0.305 lower to 0.107 lower)

Synbiotics/Probiotics compared to Control in women with PCOS

Bibliography:

Certainty assessment							Summary of findings			
**High-sensitivity c-reactive protein (follow-up: range 8 weeks to 12 weeks; assessed with: Laboratory test)**										
466 (8 RCTs)	serious[a]	very serious[c]	not serious	serious[d]	none	⨁◯◯◯ Very low[a,c,d]	228	238	-	228	MD **0.594 mg/L lower** (0.968 lower to 0.221 lower)
**Total antioxidant capacity (follow-up: range 8 weeks to 12 weeks; assessed with: Laboratory test)**										
195 (5 RCTs)	serious[a]	serious[c]	not serious	serious[d]	none	⨁◯◯◯ Very low[a,c,d]	97	98	-	97	MD **135.935 mmol/L higher** (3.201 lower to 275.07 higher)
**Malondialdehyde (follow-up: range 8 weeks to 12 weeks; assessed with: Laboratory test)**										
212 (3 RCTs)	not serious	serious[c]	not serious	serious[d]	none	⨁⨁◯◯ Low[c,d]	106	106	-	106	MD **0.095 µmol/L higher** (0.51 lower to 0.321 higher)
**Nitric oxide (follow-up: range 8 weeks to 12 weeks; assessed with: Laboratory test)**										
170 (3 RCTs)	serious[a]	very serious[c]	not serious	not serious	none	⨁◯◯◯ Very low[a,c]	85	85	-	85	MD **5.372 µmol/L higher** (1.131 higher to 9.613 higher)
**Systolic blood pressure (follow-up: range 8 weeks to 12 weeks; assessed with: Tool)**										
236 (4 RCTs)	not serious	not serious	not serious	serious[d]	none	⨁⨁⨁◯ Moderate[d]	117	119	-	117	MD **1.302 mmHg lower** (4.668 lower to 2.064 higher)
**diastolic blood pressure (follow-up: range 8 weeks to 12 weeks; assessed with: tool)**										

Synbiotics/Probiotics compared to Control in women with PCOS
Bibliography:

Certainty assessment							Summary of findings				
236<br>(4 RCTs)	not serious	not serious	not serious	not serious	none	⊕⊕⊕⊕<br>High	117	119	-	117	MD **2.218 mmHg lower**<br>(4.425 lower to 0.01 lower)

CI: confidence interval; **MD:** mean difference

Explanations

a. As per the Cochrane risk of bias assessment (Rob2), biases were identified due to issues related to the randomization and allocation concealment processes, deviations from the intended intervention, and measurement of outcomes across the studies.

b. The Begg's test in the publication bias was nonsignificant, however the Eggers' test was found to be significant showing the probable presence f a publication bias. We further used the trim-and-fill method and the number of the found articles was less than 10.

c. Based on the overall analyses, a notable level of high heterogeneity (indicated by a high I-squared value) was observed across the studies.

d. Based on the pooled analyses, the confidence intervals are wide or include a null effect, which suggests uncertainty about the true magnitude or direction of the effect.
