# Supplementary material for: The Effect of Prebiotics, Alone or as Part of Synbiotics, on Cardiometabolic Parameters in Women with Polycystic Ovary Syndrome: A Systematic Review and Meta-Analysis of Randomized Controlled Trials
Source: Biomedicines. 2025 Jan 13;13(1):177. doi: 10.3390/biomedicines13010177 (PMC11760460; doi:10.3390/biomedicines13010177)
Supplement: Supplementary file 1 [file biomedicines-13-00177-s001.zip › Table S4_PCOS_Syn_Table_Anthropometrics.pdf]

**The effect of prebiotics, alone or as part of synbiotics, on cardiometabolic parameters in women with polycystic ovary syndrome: a systematic review and meta-analysis of randomized controlled trials**

**Elham Razmpoosh [1*], Mala S. Sivanandy [2 *], Alan M. Ehrlich [3 •]**
[1] Department of Health Research Methods, Evidence and Impact (HEI), McMaster University, Hamilton, Canada.
[2] PCOS Center, Division of Endocrinology, Beth Israel Deaconess Medical Center, Harvard Medical School, Boston, USA.
[3] Department of Family Medicine and Community Health, UMass Chan Medical School, Worcester, MA and EBSCO Information Services, Ipswich MA, USA.

- **Dr. Alan M. Ehrlich, MD, FAAFP**
  Department of Family Medicine and Community Health, UMass Chan Medical School, Worcester MA, and EBSCO Information Services, USA
  **Tel**: +1-508-439-1157
  **Email**: aehrlich@ebsco.com
  **Orchid ID**: 0009-0002-6052-9902

[*] Elham Razmpoosh and Mala S. Sivanandy contributed equally to this work.

**Supplementary Table S4** Meta-analysis showing the effect of prebiotics and synbiotics interventions on anthropometric indices (all analyses were conducted using a random-effects model).

| Outcomes | Meta-analysis | | | | | Heterogeneity | | | |
|---|---|---|---|---|---|---|---|---|---|
| | Study group | Number of studies | Number of participants | WMD (95% CI) | *P* effect | Q statistic | *P* within group [1] | *I*-squared (%) | *P* between group [2] |
| **BMI ($kg/m^2$)** | Overall | 14 | 852 | -0.510 (-0.669, -0.351) | **<0.001** | 3.86 | 0.996 | 0.0 | - |
| **Weight (kg)** | Overall | 14 | 852 | -1.857 (2.464, -1.249) | **<0.001** | 15.51 | 0.344 | 9.8 | - |
| **WC (cm)** | Overall | 9 | 665 | -3.11 (-4.193, -2.028) | **<0.001** | 38.47 | < 0.001 | 76.6 | - |
| | **DASH diet** | | | | | | | | |
| | Yes | 3 | 153 | -3.154 (-5.2, -1.109) | **<0.001** | 26.28 | <0.001 | 92.4 | 0.106 |
| | No | 6 | 412 | -3.009 (-4.474, -1.544) | **<0.001** | 9.33 | 0.156 | 35.7 | |
| | **LC diet** | | | | | | | | |
| | Yes | 3 | 142 | -3.839 (-5.209, -2.468) | **<0.001** | 15.97 | <0.001 | 87.5 | 0.024 |

| | | | | | | | | | |
|---|---|---|---|---|---|---|---|---|---|
| | No | 6 | 423 | -2.660 ( -3.539, -1.782) | **<0.001** | 3.66 | 0.723 | 0.0 | |
| | **Baseline BMI** | | | | | | | | |
| | Obesity (BMI≥30 kg/m$^2$) | 5 | 272 | -3.345 (-4.614, -2.076) | **<0.001** | 30.42 | <0.001 | 86.9 | 0.127 |
| | Overweight (BMI between 25-29.9 kg/m$^2$) | 4 | 293 | -2.379(-4.104, -0.655) | **0.007** | 2.09 | 0.720 | 0.0 | |
| **HC (cm)** | Overall | 7 | 463 | -1.203 (-2.992, 0.586) | 0.188 | 21.90 | 0.003 | 68.0 | - |
| | Overall (excluding a study by Asemi 2015) | 6 | 415 | -0.189 (-1.445, 1.067) | 0.768 | 8.08 | 0.232 | 25.8 | - |
| | **Type of interventions** | | | | | | | | |
| | Prebiotics | 3 | 185 | -3.154 (-6.885, 0.577) | 0.098 | 12.04 | 0.002 | 83.4 | 0.006 |
| | Synbiotics | 5 | 330 | 0.516 (-0.626, 1.657) | 0.382 | 2.31 | 0.804 | 0.0 | |
| | **DASH diet** | | | | | | | | |
| | Yes | 2 | 103 | -2.456 (-7.078, 2.167) | 0.298 | 9.96 | 0.002 | 90.0 | 0.036 |
| | No | 6 | 412 | 0.178 (-0.929, 1.286) | 0.752 | 7.98 | 0.239 | 24.9 | |
| | **LC diet** | | | | | | | | |
| | Yes | 3 | 142 | -1.143 (-4.345, 2.059) | 0.484 | 17.47 | 0.000 | 88.5 | 0.513 |
| | No | 5 | 384 | -0.733 (-2.224, 0.758) | 0.335 | 4.79 | 0.442 | 0.0 | |

| | | | | | | | | | |
|---|---|---|---|---|---|---|---|---|---|
| | **Type of Prebiotics** | | | | | | | | |
| | Other (Psyllium, Fiber) | 3 | 165 | -3.154 (-6.885 0.577 | 0.098 | 12.04 | 0.002 | 83.4 | 0.006 |
| | Inulin | 5 | 350 | 0.516 (-0.626 1.657 | 0.376 | 2.29 | 0.804 | 0.0 | |
| | **Baseline BMI** | | | | | | | | |
| | Obesity (BMI≥30 kg/m$^2$) | 4 | 222 | -0.965 -3.577 1.646 | 0.469 | 17.47 | 0.001 | 82.8 | 0.406 |
| | Overweight (BMI between 25-29.9 kg/m$^2$) | 4 | 293 | -0.905 (-2.712, 0.903) | 0.327 | 4.74 | 0.316 | 15.5 | |
| **WHR** | Overall | 6 | 401 | -0.015 (-0.026 , -0.003) | **0.011** | 11.88 | 0.065 | 49.5 | - |
| | Overall (excluding a study by Chudzicka-Strugala 2021) | 5 | 362 | -0.011 (-0.020 , -0.001) | 0.028 | 6.77 | 0.238 | 26.2 | - |
| | **Type of intervention** | | | | | | | | |
| | Prebiotics | 2 | 103 | -0.004 (-0.014, 0.005) | 0.346 | 0.11 | 0.737 | 0.0 | 0.018 |
| | Synbiotics | 4 | 298 | -0.021 (-0.034, -0.008) | 0.002 | 6.17 | 0.187 | 35.2 | |
| | **LC diet** | | | | | | | | |
| | Yes | 2 | 94 | -0.020 (-0.055, 0.014) | 0.239 | 6.31 | 0.012 | 84.2 | 0.462 |
| | No | 4 | 307 | -0.014 (-0.026, -0.002) | **0.023** | 5.03 | 0.284 | 20.5 | |
| | **Type of Prebiotics** | | | | | | | | |

| | | | | | | | | | |
|---|---|---|---|---|---|---|---|---|---|
| | Other (Psyllium, Fiber) | 2 | 103 | -0.004 (-0.014, 0.005) | 0.346 | 0.11 | 0.737 | 0.0 | 0.018 |
| | Inulin | 4 | 298 | -0.021 (-0.034, -0.008) | 0.002 | 6.17 | 0.187 | 35.2 | |
| | **Duration of Intervention** | | | | | | | | |
| | 12 weeks | 3 | 193 | -0.013 (-0.035, 0.008) | 0.212 | 6.79 | 0.034 | 70.5 | 0.199 |
| | 8 weeks | 3 | 208 | -0.018 (-0.030, -0.005) | **0.007** | 3.45 | 0.328 | 13.0 | |
| | **Baseline BMI** | | | | | | | | |
| | Obesity (BMI≥30 kg/m$^2$) | 3 | 193 | -0.013 (-0.035, 0.008) | 0.212 | 6.79 | 0.034 | 70.5 | 0.199 |
| | Overweight (BMI between 25-29.9 kg/m$^2$) | 3 | 208 | -0.018 (-0.030, -0.005) | **0.007** | 3.45 | 0.328 | 13.0 | |
| **Fat Mass (kg)** | Overall | 4 | 202 | -1.496 (-2.205, -0.787) | <0.001 | 7.93 | 0.047 | 62.2 | - |
| | **Country** | | | | | | | | |
| | Iran | 2 | 103 | -1.066 (-1.836, -0.296) | **0.007** | 0.29 | 0.589 | 0.0 | 0.505 |
| | Other countries | 2 | 99 | -1.827, -3.181, -0.474) | 0.008 | 7.19 | 0.007 | 86.1 | |
| | **Duration of Intervention** | | | | | | | | |
| | 12 weeks | 2 | 94 | -1.194 (-1.490, -0.899) | <0.001 | 0.07 | 0.798 | 0.0 | 0.010 |
| | 8 weeks | 2 | 108 | -2.199 (-4.142, -0.255) | 0.027 | 1.29 | 0.257 | 22.2 | |

[1] Calculated from a random-effects model

[2] Calculated from a fixed-effect model

Abbreviations**:** DASH, dietary approaches to stop hypertension; LC, low-calorie; HC, hip circumference; WC, waist circumference; WHR, waist-to-hip ratio; BMI, body mass index; WMD, weighted mean difference.

(Negative signs in WMD indicate a negative difference in the outcome).
