# Supplementary material for: The Effect of Prebiotics, Alone or as Part of Synbiotics, on Cardiometabolic Parameters in Women with Polycystic Ovary Syndrome: A Systematic Review and Meta-Analysis of Randomized Controlled Trials
Source: Biomedicines. 2025 Jan 13;13(1):177. doi: 10.3390/biomedicines13010177 (PMC11760460; doi:10.3390/biomedicines13010177)
Supplement: Supplementary file 1 [file biomedicines-13-00177-s001.zip › Table S5_PCOS_Syn_If.OX.pdf]

The effect of prebiotics, alone or as part of synbiotics, on cardiometabolic parameters in women with polycystic ovary syndrome: a systematic review and meta-analysis of randomized controlled trials

Elham Razmpoosh [1*], Mala S. Sivanandy [2*], Alan M. Ehrlich [3] •
[1] Department of Health Research Methods, Evidence and Impact (HEI), McMaster University, Hamilton, Canada.
[2] PCOS Center, Division of Endocrinology, Beth Israel Deaconess Medical Center, Harvard Medical School, Boston, USA.
[3] Department of Family Medicine and Community Health, UMass Chan Medical School, Worcester, MA and EBSCO Information Services, Ipswich MA, USA.

- **Dr. Alan M. Ehrlich, MD, FAAFP**
  Department of Family Medicine and Community Health, UMass Chan Medical School, Worcester MA, and EBSCO Information Services, USA
  Tel: +1-508-439-1157
  Email: aehrlich@ebsco.com
  Orchid ID: 0009-0002-6052-9902

[*] Elham Razmpoosh and Mala S. Sivanandy contributed equally to this work.

Supplementary Table S5 Meta-analysis showing the effect of prebiotics and synbiotics interventions on CRP, TAC, NO, and blood pressure parameters (all analyses were conducted using a random-effects model).

Outcomes	Meta-analysis					Heterogeneity			
	**Study group**	**Number of studies**	**Number of participants**	**WMD (95% CI) (kg)**	***P* effect**	**Q statistic**	***P* within group [1]**	***I*-squared (%)**	***P* between group [2]**
**hs-CRP (mg/dL)**	Overall	8	466	-0.594 (-0.968, -0.221)	0.002	190.09	<0.001	96.3	-
	**Type of Prebiotics**								
	Other (Psyllium, Fiber)	3	296	-1.434 (-1.642, -1.227)	<0.001	0.72	0.696	0.0	<0.001
	Inulin	5	252	-0.030 (-0.163, 0.103)	0.663	13.59	0.009	70.6	
	**Baseline BMI**								
	Obesity (BMI≥30 kg/m^2)	2	147	-1.433 (-1.642 , -1.225)	<0.001	0.22	0.641	0.0	<0.001
	Overweight (BMI between 25-29.9 kg/m^2)	6	319	-0.045 (-0.190, 0.100)	0.545	16.88	0.005	70.4	

**TAC** (mmol/L)	Overall	4	195	135.935 (-3.201, 275.070)	0.056	13.24	0.004	77.3	-
**NO** (μmol/L)	Overall	3	170	5.372 1.131, 9.613)	0.013	15.63	<0.001	87.2	-
**SBP (mmHg)**	Overall	3	236	-1.302 (-4.668, 2.064)	0.448	4.37	0.224	31.4	-
**DBP (mmHg)**	Overall	3	236	-2.218 (-4.425 , -0.010)	0.049	0.82	0.845	0.0	-

[1] Calculated from a random-effects model

[2] Calculated from a fixed-effect model

Abbreviations**:** hs-CRP, High-Sensitivity C-Reactive Protein; TAC, Total Antioxidant Capacity; NO, Nitric Oxide; SBP, Systolic Blood Pressure; DBP, Diastolic Blood Pressure; BMI, body mass index; WMD, weighted mean difference.

(Negative signs in WMD indicate a negative difference in the outcome).
