# Supplementary material for: The Effect of Prebiotics, Alone or as Part of Synbiotics, on Cardiometabolic Parameters in Women with Polycystic Ovary Syndrome: A Systematic Review and Meta-Analysis of Randomized Controlled Trials
Source: Biomedicines. 2025 Jan 13;13(1):177. doi: 10.3390/biomedicines13010177 (PMC11760460; doi:10.3390/biomedicines13010177)
Supplement: Supplementary file 1 [file biomedicines-13-00177-s001.zip › Table S6_PCOS_Syn_Lipids.pdf]

The effect of prebiotics, alone or as part of synbiotics, on cardiometabolic parameters in women with polycystic ovary syndrome: a systematic review and meta-analysis of randomized controlled trials

Elham Razmpoosh [1*], Mala S. Sivanandy [2*], Alan M. Ehrlich [3] •

[1] Department of Health Research Methods, Evidence and Impact (HEI), McMaster University, Hamilton, Canada.

[2] PCOS Center, Division of Endocrinology, Beth Israel Deaconess Medical Center, Harvard Medical School, Boston, USA.

[3] Department of Family Medicine and Community Health, UMass Chan Medical School, Worcester, MA and EBSCO Information Services, Ipswich MA, USA.

- **Dr. Alan M. Ehrlich, MD, FAAFP**
  Department of Family Medicine and Community Health, UMass Chan Medical School, Worcester MA, and EBSCO Information Services, USA
  Tel: +1-508-439-1157
  Email: aehrlich@ebsco.com
  Orchid ID: 0009-0002-6052-9902

[*] Elham Razmpoosh and Mala S. Sivanandy contributed equally to this work.

Supplementary Table S6 Meta-analysis showing the effect of prebiotics and synbiotics interventions on lipid profile (all analyses were conducted using a random-effects model).

Outcomes	Meta-analysis					Heterogeneity			
	Study group	Number of studies	Number of participants	WMD (95% CI)	*P* effect	Q statistic	*P* within group [1]	*I*-squared (%)	*P* between group [2]
HDL (mg/dL)	Overall	10	621	2.152 (-0.028, 4.277)	0.577	24.00	0.005	59.9	-
	**Duration of Intervention**								
	8 week	4	251	3.998 (1.271, 6.725)	0.004	3.43	0.488	0.0	0.009
	12 weeks	6	370	1.154 (-1.472, 3.780)	0.389	15.75	0.008	67.9	
	**Baseline BMI**								
	Obesity (BMI≥30 kg/m^2)	3	186	-1.39 (-3.042, 0.259)	0.099	0.24	0.888	0.0	<0.001
	Overweight (BMI between 25-29.9 kg/m^2)	7	435	3.757 (1.870, 5.645)	0.001	7.67	0.363	8.7	
LDL (mg/dL)	Overall	10	621	-10.149 (-17.168, -3.129)	0.005	36.67	<0.001	72.7	-

	**Type of interventions**								
	Prebiotics	4	208	-22.8631 (-39.862, -5.758)	0.009	14.4	0.002	79.2	<0.001
	Synbiotics	6	413	-2.622 (-6.835, 1.590)	0.222	7.36	0.288	18.5	
	**Type of Prebiotics**								
	Other (Psyllium, Fiber)	3	158	-30.691 (-40.862, -20.519)	<0.001	1.31	0.518	0.0	<0.001
	Inulin	7	463	-2.776 (-6.082, 0.530)	0.100	7.36	0.357	9.4	
	**Duration**								
	8 weeks	4	251	-20.160 (-30.452, -9.868)	<0.001	4.27	0.370	6.4	0.001
	12 weeks	6	370	-6.012 (-13.466, 1.442)	0.114	21.20	0.001	76.7	
	**Baseline BMI**								
	Obesity (BMI≥30 kg/m^2)	3	186	-3.496 (-9.045, 2.054)	0.217	3.16	0.206	36.7	0.007
	Overweight (BMI between 25-29.9 kg/m^2)	7	435	-12.717 (-23.877, -1.547)	0.026	26.24	<0.001	74.7	
**TC (mg/dL)**	Overall	10	621	-8.563 (-13.720, -3.406)	0.001	16.89	0.077	40.8	-
	Overall (excluding a study by Gholizadeh-Shamasbi 2019)	9	559	-6.253 (-8.917, -3.590)	<0.001	6.93	0.544	0.0	-
	**Type of intervention**								

	Prebiotics	4	208	-15.647 (-27.548, -3.747)	0.010	4.56	0.117	49.1	0.024
	Synbiotics	6	413	-5.730 (-9.775, -1.685)	0.005	6.75	0.345	11.0	
	**LC diet**								
	Yes	2	87	-6.082 (-9.185 , -2.979)	<0.001	0.11	0.738	0.0	0.498
	No	8	504	-9.805 (-17.179, -2.430)	0.009	15.81	0.043	49.8	
	**Type of Prebiotics**								
	Other (Psyllium, Fiber)	3	158	-18.488 (-36.083, -0.894)	0.039	4.56	0.102	56.2	0.024
	Inulin	7	463	-6.234 (-9.579, -2.888)	0.005	7.75	0.381	6.4	
	**Duration of Intervention**								
	8 weeks	4	251	-11.248 (-20.024, -2.472)	0.012	3.34	0.502	0.0	0.274
	12 weeks	6	370	-7.739 (-14.572, -0.906)	0.026	11.84	0.029	60.0	
	**Baseline BMI**								
	Obesity (BMI≥30 kg/m^2)	3	186	-5.673 (-8.662 , -2.684)	<0.001	1.04	0.595	0.0	0.14
	Overweight (BMI between 25-29.9 kg/m^2)	7	435	-11.499 (-19.505, -3.493)	0.005	13.16	0.0681	46.9	

**TG (mg/dL)**	Overall	10	621	-13.974 (-22.452, -5.590)	0.001	15.12	0.128	33.8	-

[1] Calculated from a random-effects model

[2] Calculated from a fixed-effect model

Abbreviations: HDL, high-density of lipoprotein cholesterol, LDL, low-density of lipoprotein cholesterol, TG, triglycerides, TC, total cholesterol; LC, low-calorie; BMI, body mass index; WMD, weighted mean difference.

(Negative signs in WMD indicate a negative difference in the outcome).
