# Supplementary material for: The Effect of Prebiotics, Alone or as Part of Synbiotics, on Cardiometabolic Parameters in Women with Polycystic Ovary Syndrome: A Systematic Review and Meta-Analysis of Randomized Controlled Trials
Source: Biomedicines. 2025 Jan 13;13(1):177. doi: 10.3390/biomedicines13010177 (PMC11760460; doi:10.3390/biomedicines13010177)
Supplement: Supplementary file 1 [file biomedicines-13-00177-s001.zip › Table S7_PCOS_Syn_Glycmic.pdf]

The effect of prebiotics, alone or as part of synbiotics, on cardiometabolic parameters in women with polycystic ovary syndrome: a systematic review and meta-analysis of randomized controlled trials

Elham Razmpoosh [1*], Mala S. Sivanandy [2*], Alan M. Ehrlich [3] •
[1] Department of Health Research Methods, Evidence and Impact (HEI), McMaster University, Hamilton, Canada.
[2] PCOS Center, Division of Endocrinology, Beth Israel Deaconess Medical Center, Harvard Medical School, Boston, USA.
[3] Department of Family Medicine and Community Health, UMass Chan Medical School, Worcester, MA and EBSCO Information Services, Ipswich MA, USA.

- **Dr. Alan M. Ehrlich, MD, FAAFP**
  Department of Family Medicine and Community Health, UMass Chan Medical School, Worcester MA, and EBSCO Information Services, USA
  Tel: +1-508-439-1157
  Email: aehrlich@ebsco.com
  Orchid ID: 0009-0002-6052-9902

[*] Elham Razmpoosh and Mala S. Sivanandy contributed equally to this work.

Supplementary Table S7 Meta-analysis showing the effect of prebiotics and synbiotics interventions on Glycemic indices (all analyses were conducted using a random-effects model).

Outcomes	Meta-analysis					Heterogeneity			
	**Study group**	**Number of studies**	**Number of participants**	**WMD (95% CI)**	***P* effect**	**Q statistic**	***P* within group [1]**	***I*-squared (%)**	***P* between group [2]**
**FPG** (mg/dL)	Overall	10	636	-4.142 (-7.233, -1.051)	0.009	174.37	<0.001	94.3	-
	**LC diet**								
	Yes	4	207	-3.616 (-8.972, 1.741)	0.186	152.35	<0.001	98.0	0.121
	No	7	479	-4.001 (-6.628, -1.074)	0.003	19.61	0.003	66.5	
	**Duration of Intervention**								
	8 weeks	5	316	-5.287 (-7.453, -3.121)	<0.001	15.17	0.010	67.0	<0.001
	12 weeks	6	370	-3.942 (-6.258, 0.280)	0.073	37.60	<0.001	87.3	
**Fasting insulin** (mg/dL)	Overall	10	624	-2.816 (-4.203, -1.429)	<0.001	61.14	<0.001	83.7	-

	**Country**								
	Iran	8	525	-3.649 (-4.641 (-2.565)	<0.001	13.72	0.083	42.7	<0.001
	Other countries	2	99	-0.563 (-1.339, 0.214)	0.155	0.40	0.525	0.0	
	**DASH diet**								
	Yes	2	108	-4.679 (-5.521, -3.837)	<0.001	0.57	0.450	0.0	<0.001
	No	8	516	-2.404 (-3.704, -1.104)	<0.001	25.97	0.001	71.3	
	**LC diet**								
	Yes	4	207	-2.224 (-4.858, 0.409)	0.098	50.62	<0.001	94.1	0.149
	No	6	479	-3.270 (-4.424, -2.115)	<0.001	8.43	0.202	29.7	
**Insulin resistance (HOMA-IR)**	Overall	9	585	-0.417 (-0.971, 0.137)	0.140	128.86	<0.001	93.0	-
	**DASH diet**								
	Yes	2	108	-1.195 (-1.483, -0.908)	<0.001	1.16	0.282	13.8	<0.001
	No	7	427	-0.249 (-0.971, 0.137)	0.400	61.07	<0.001	88.5	
**Insulin sensitivity (QUICKI)**	Overall	7	457	0.0186 (0.007, 0.026)	0.001	86.70	<0.001	91.9	-
	**Duration of intervention**								
	8 weeks	4	269	0.013 (0.002, 0.024)	0.018	71.74	<0.001	94.4	<0.001

	12 weeks	3	188	0.025 (0.017, 0.033)	<0.001	1.40	0.496	0.0	

[1] Calculated from a random-effects model

[2] Calculated from a fixed-effect model

Abbreviations: FPG, fasting plasma glucose, HOMA-IR, Homeostasis Model Assessment for Insulin Resistance, QUICKI, Quantitative Insulin Sensitivity Check Index; DASH, dietary approaches to stop hypertension; LC, low-calorie; BMI, body mass index; WMD, weighted mean difference.

(Negative signs in WMD indicate a negative difference in the outcome).
