# Supplementary material for: The Effect of Prebiotics, Alone or as Part of Synbiotics, on Cardiometabolic Parameters in Women with Polycystic Ovary Syndrome: A Systematic Review and Meta-Analysis of Randomized Controlled Trials
Source: Biomedicines. 2025 Jan 13;13(1):177. doi: 10.3390/biomedicines13010177 (PMC11760460; doi:10.3390/biomedicines13010177)
Supplement: Supplementary file 1 [file biomedicines-13-00177-s001.zip › Table S8_PCOS_Syn_BP.pdf]

The effect of prebiotics, alone or as part of synbiotics, on cardiometabolic parameters in women with polycystic ovary syndrome: a systematic review and meta-analysis of randomized controlled trials

Elham Razmpoosh [1*], Mala S. Sivanandy [2*], Alan M. Ehrlich [3] •
[1] Department of Health Research Methods, Evidence and Impact (HEI), McMaster University, Hamilton, Canada.
[2] PCOS Center, Division of Endocrinology, Beth Israel Deaconess Medical Center, Harvard Medical School, Boston, USA.
[3] Department of Family Medicine and Community Health, UMass Chan Medical School, Worcester, MA and EBSCO Information Services, Ipswich MA, USA.

- **Dr. Alan M. Ehrlich, MD, FAAFP**
  Department of Family Medicine and Community Health, UMass Chan Medical School, Worcester MA, and EBSCO Information Services, USA
  Tel: +1-508-439-1157
  Email: aehrlich@ebsco.com
  Orchid ID: 0009-0002-6052-9902

[*] Elham Razmpoosh and Mala S. Sivanandy contributed equally to this work.

Supplementary Table S8 Meta-analysis showing the effect of prebiotics and synbiotics interventions on blood pressure parameters (all analyses were conducted using a random-effects model).

Outcomes	Meta-analysis					Heterogeneity			
	Study group	Number of studies	Number of participants	WMD (95% CI) (kg)	*P* effect	Q statistic	*P* within group [1]	*I*-squared (%)	*P* between group [2]
**SBP (mmHg)**	Overall	3	236	-1.302 (- 4.668, 2.064)	0.448	4.37	0.224	31.4	-
**DBP (mmHg)**	Overall	3	236	-2.218 (- 4.425 , - 0.010)	0.049	0.82	0.845	0.0	-

[1] Calculated from a random-effects model

[2] Calculated from a fixed-effect model

Abbreviations: SBP, Systolic Blood Pressure; DBP, Diastolic Blood Pressure; BMI, body mass index; WMD, weighted mean difference.

(Negative signs in WMD indicate a negative difference in the outcome).
