# Supplementary material for: The Effect of Prebiotics, Alone or as Part of Synbiotics, on Cardiometabolic Parameters in Women with Polycystic Ovary Syndrome: A Systematic Review and Meta-Analysis of Randomized Controlled Trials
Source: Biomedicines. 2025 Jan 13;13(1):177. doi: 10.3390/biomedicines13010177 (PMC11760460; doi:10.3390/biomedicines13010177)
Supplement: Supplementary file 1 [file biomedicines-13-00177-s001.zip › Table S9_PCOS_Syn_Hormonal levels.pdf]

The effect of prebiotics, alone or as part of synbiotics, on cardiometabolic parameters in women with polycystic ovary syndrome: a systematic review and meta-analysis of randomized controlled trials

Elham Razmpoosh [1*], Mala S. Sivanandy [2*], Alan M. Ehrlich [3•]

[1] Department of Health Research Methods, Evidence and Impact (HEI), McMaster University, Hamilton, Canada.

[2] PCOS Center, Division of Endocrinology, Beth Israel Deaconess Medical Center, Harvard Medical School, Boston, USA.

[3] Department of Family Medicine and Community Health, UMass Chan Medical School, Worcester, MA and EBSCO Information Services, Ipswich MA, USA.

- **Dr. Alan M. Ehrlich, MD, FAAFP**
  Department of Family Medicine and Community Health, UMass Chan Medical School, Worcester MA, and EBSCO Information Services, USA
  Tel: +1-508-439-1157
  Email: aehrlich@ebsco.com
  Orchid ID: 0009-0002-6052-9902

[*] Elham Razmpoosh and Mala S. Sivanandy contributed equally to this work.

Supplementary Table S9 Meta-analysis showing the effect of prebiotics and synbiotics interventions on Hormonal levels (all analyses were conducted using a random-effects model).

Outcome	Meta-analysis					Heterogeneity			
	Study group	Number of studies	Number of participants	WMD (95% CI) (IU/L)	*P* effect	Q statistic	*P* within group [1]	*I*-squared (%)	*P* between group [2]
FSH	Overall	4	191	0.102 (-0.475, 1.564)	<0.001	3.22	0.36	6.7	-
	**LC diet**								
	No	2	99	-0.349 (-2.235, 1.536)	0.716	0.12	0.732	0.0	0.03
	Yes	2	92	1.177 (0.835, 1.519)	<0.001	0.66	0.416	0.0	
LH	Overall	4	191	1.05 (-0.392, 2.493)	0.153	8.96	0.030	66.5	-
	**LC diet**								
	No	2	99	0.738 (-1.204, 2.680)	0.457	0.16	0.687	0.0	0.209
	Yes	2	92	1.150 (-1.095, 3.395)	0.315	7.71	0.005	87.0	

**Total testostrone**	Overall	7	416	−0.268, (−0.473,−0.063)	0.01	35.65	0.001	80.4	-
	**Country**								
	Iran	5	267	-0.324 (-0.502, -0.145)	<0.001	10.71	0.057	53.3	0.002
	Other counries	2	99	-0.156 (-0.747, 0.435)	0.606	11.63	0.001	91.4	
	**Type of intervention**								
	Synbiotics	4	196	-0.312 (-0.463, -0.160)	<0.001	1.78	0.620	0.0	0.022
	Probiotics	3	220	-0.208 (-0.562, 0.146)	0.249	30.50	<0.001	90.2	
	**LC diet**								
	No	3	152	-0.263 (-0.435, -0.09)	0.003	0.44	0.803	0.0	0.19
	Yes	4	214	-0.137 (-0.430, 0.156)	0.360	21.35	<0.001	86.0	
	**Type of Prebiotics**								
	Inulin	4	196	-0.312 (-0.463, -0.160)	<0.001	1.78	0.249	0.0	0.022
	Other (Psyllium, Fiber)	3	220	-0.208 (-0.562, 0.146)	0.249	30.50	<0.001	90.2	
	**Baseline BMI**								
	Obesity (BMI≥30 kg/m^2)	3	154	-0.282 (-0.496, -0.067)	0.01	3.27	0.195	38.8	0.004
	Overweight (BMI between 25-29.9 kg/m^2)	4	262	-0.286 (-0.614, 0.042)	0.087	28.81	0.000	85.8	

**SHBG**	Overall	6	324	13.733 (-0.095, 27.560)	0.052	230.07	<0.001	97.8	-
	**Country**								
	Iran	4	225	11.453 (6.825, 16.080)	<0.000	4.04	0.257	25.7	<0.001
	Other countries	2	99	15.313 (-23.503, 54.130)	0.439	180.19	<0.001	99.4	
**DHEAS**	Overall	3	161	0.179 (-0.381, 0.739)	0.533	5.76	0.056	65.3	-
**FAI**	Overall	5	285	-0.227 (-0.305, -0.148)	<0.000	10.04	0.04	60.2	-
**Hirsutism**	Overall	3	149	-1.128 (12.286, 0.03)	0.056	21.84	<0.001	90.8	

[1] Calculated from a random-effects model

[2] Calculated from a fixed-effect model

Abbreviations**:** FSH, follicle stimulating hormone; LH, luteinizing hormone; SHBG, Sex Hormone-Binding Globulin; DHEAS, Dehydroepiandrosterone sulfate; FAI, Free androgen index; LC, low-calorie; BMI, body mass index; WMD, weighted mean difference.

(Negative signs in WMD indicate a negative difference in the outcome).
