# Supplementary figures and images for: The Effect of Prebiotics, Alone or as Part of Synbiotics, on Cardiometabolic Parameters in Women with Polycystic Ovary Syndrome: A Systematic Review and Meta-Analysis of Randomized Controlled Trials
Source: Biomedicines. 2025 Jan 13;13(1):177. doi: 10.3390/biomedicines13010177 (PMC11760460; doi:10.3390/biomedicines13010177)

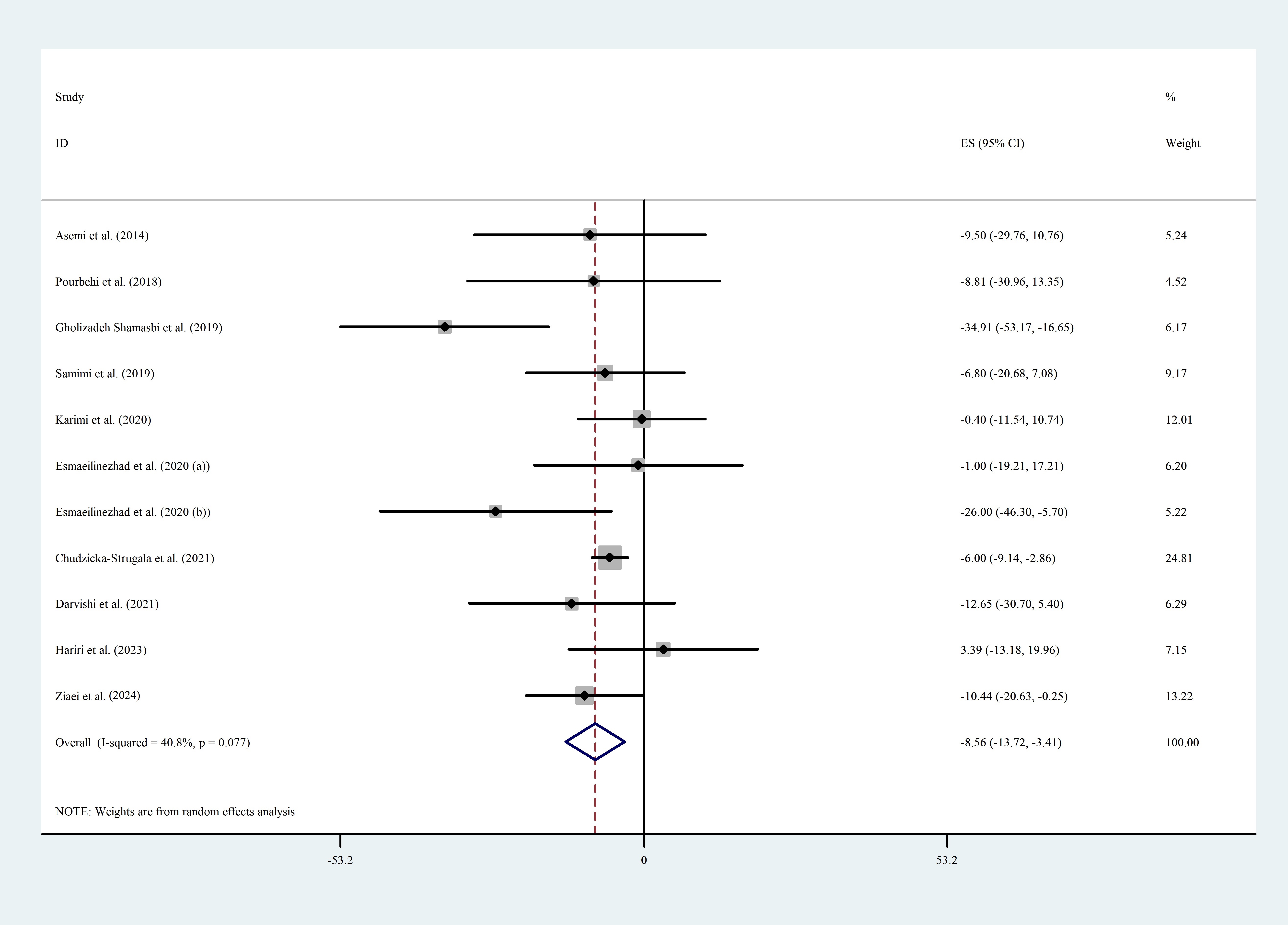

Supplement: Supplementary file 1 [file biomedicines-13-00177-s001.zip › Figure S10_TC .jpg]

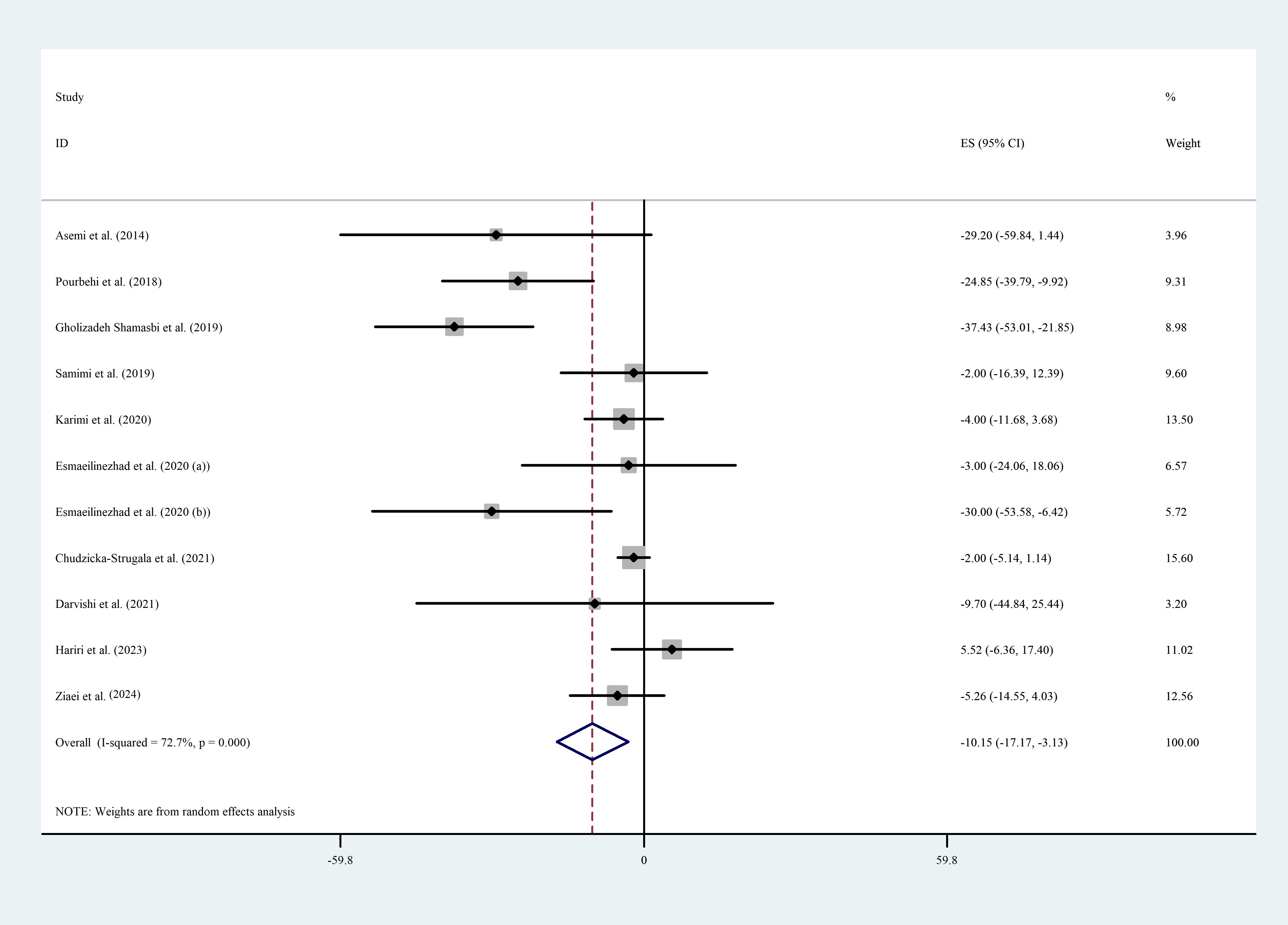

Supplement: Supplementary file 1 [file biomedicines-13-00177-s001.zip › Figure S11_LDL .jpg]

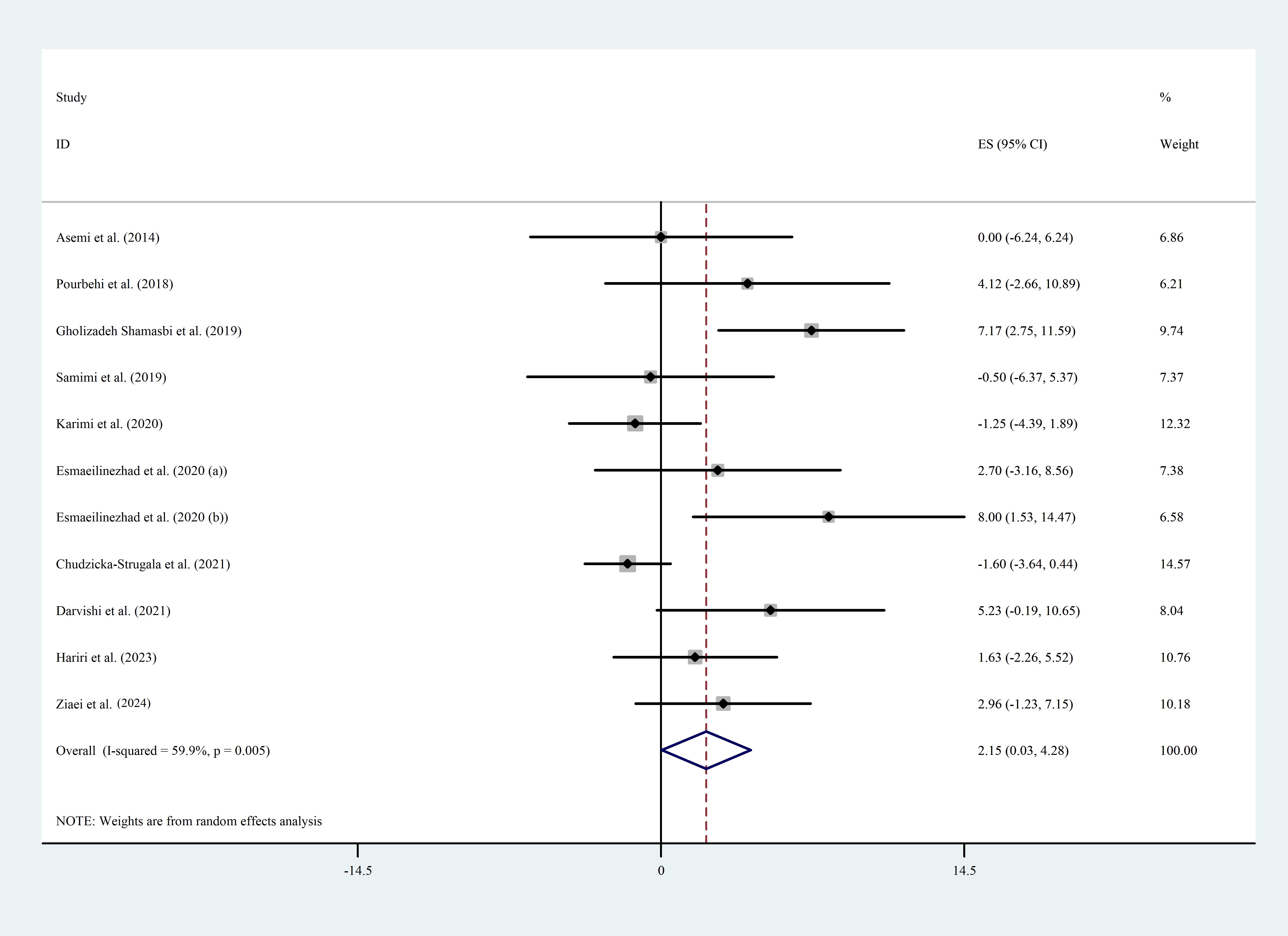

Supplement: Supplementary file 1 [file biomedicines-13-00177-s001.zip › Figure S12_HDL .jpg]

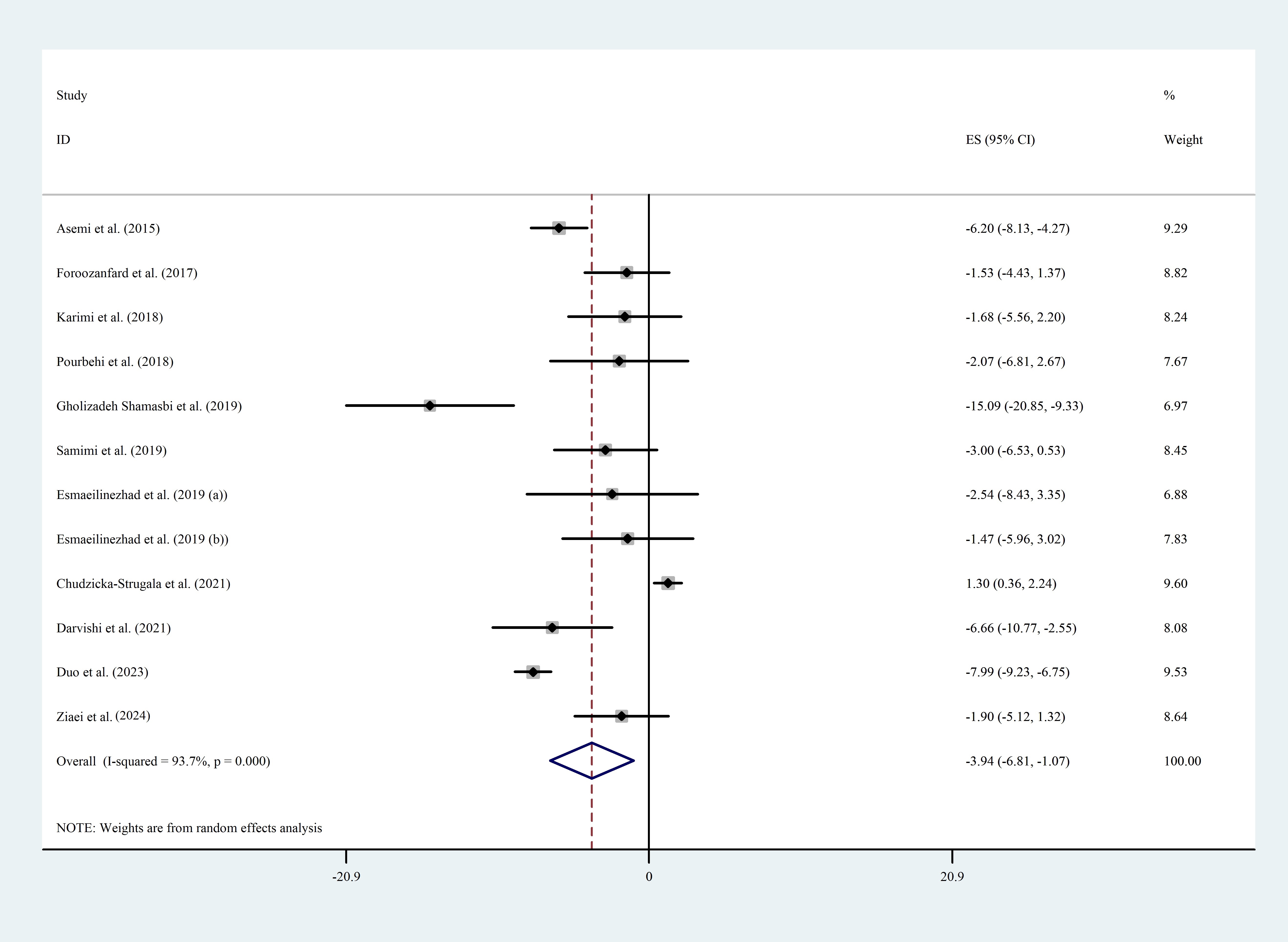

Supplement: Supplementary file 1 [file biomedicines-13-00177-s001.zip › Figure S13_FPG .jpg]

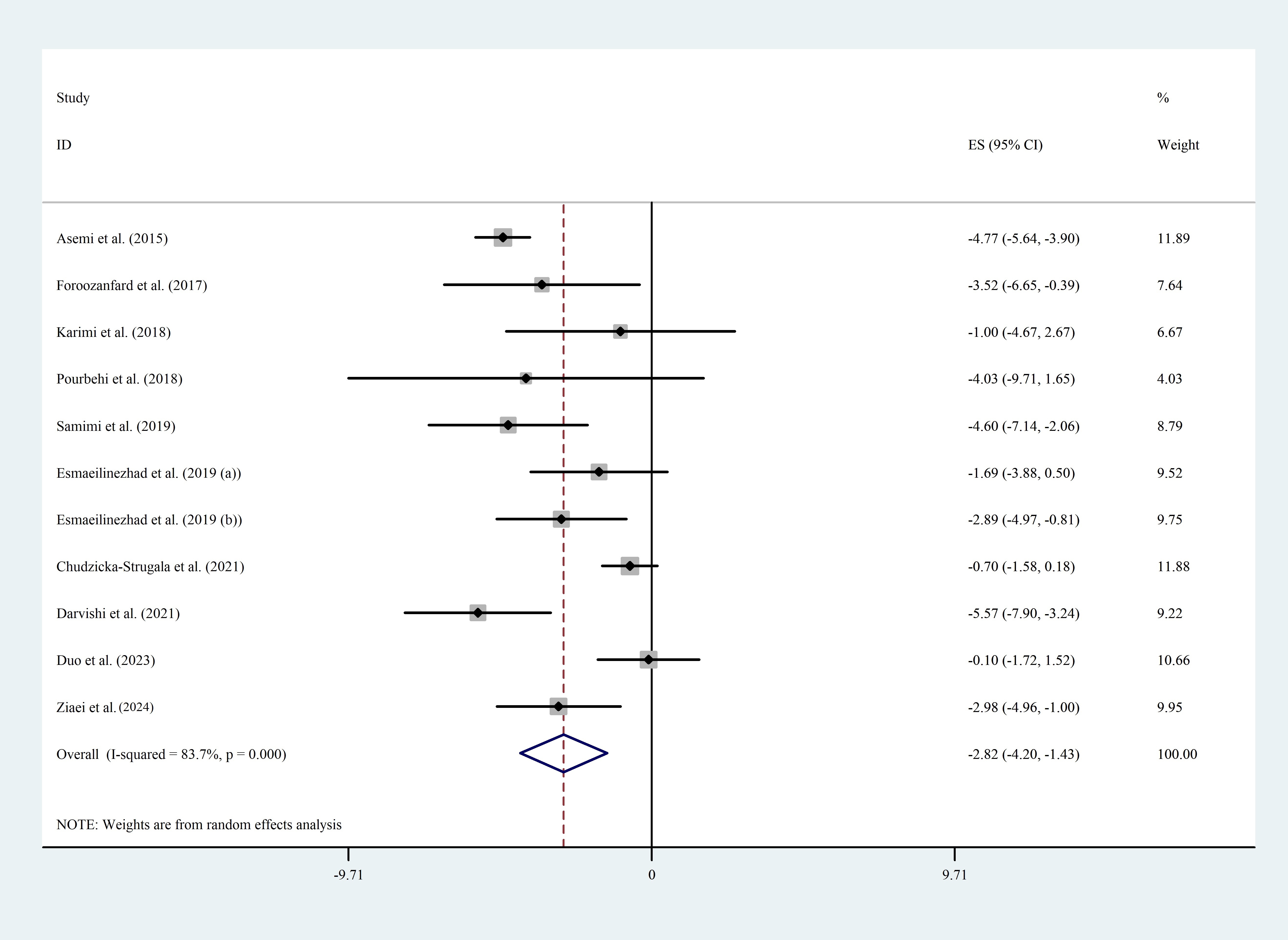

Supplement: Supplementary file 1 [file biomedicines-13-00177-s001.zip › Figure S14_insulin .jpg]

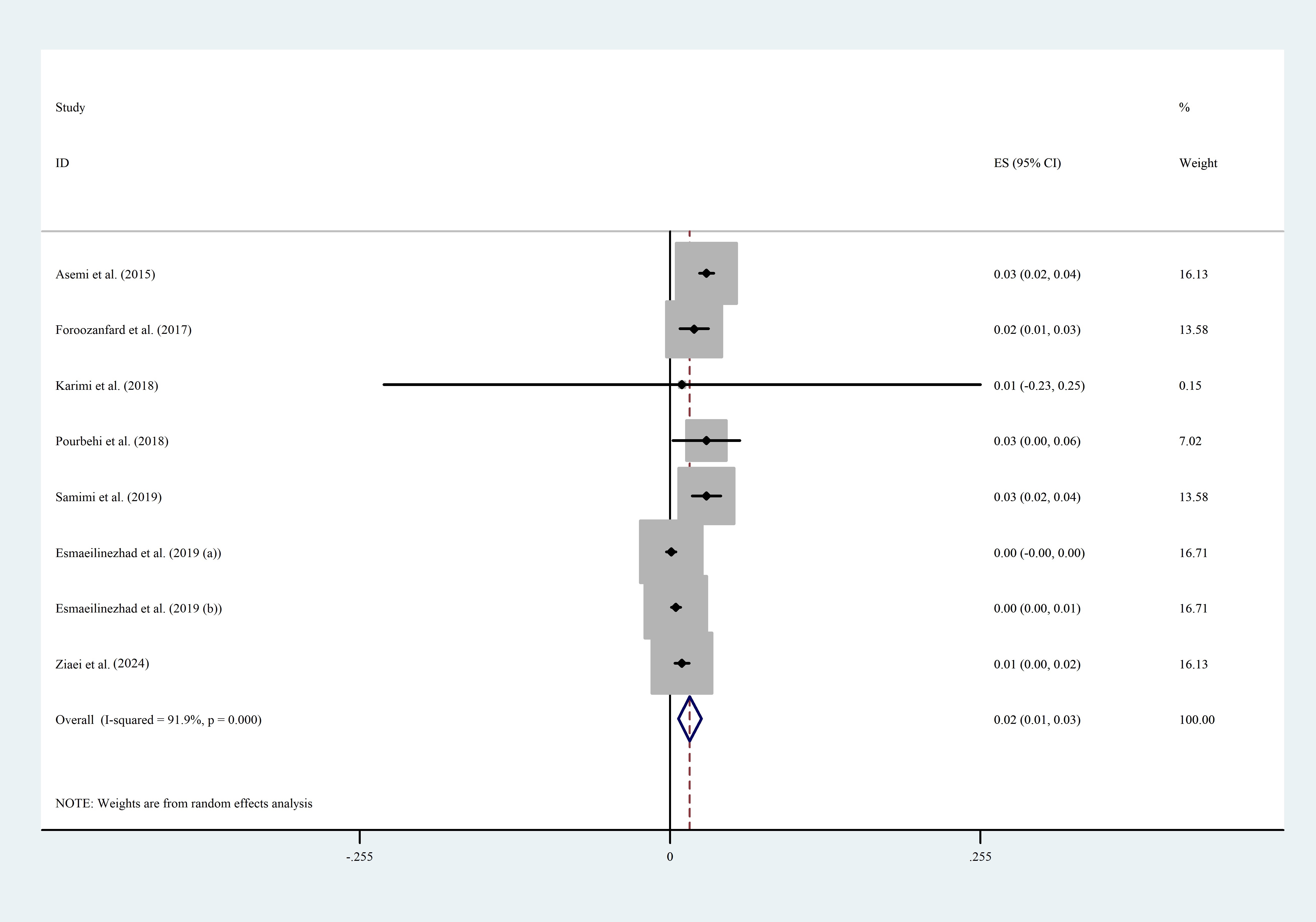

Supplement: Supplementary file 1 [file biomedicines-13-00177-s001.zip › Figure S15_QUICKI .jpg]

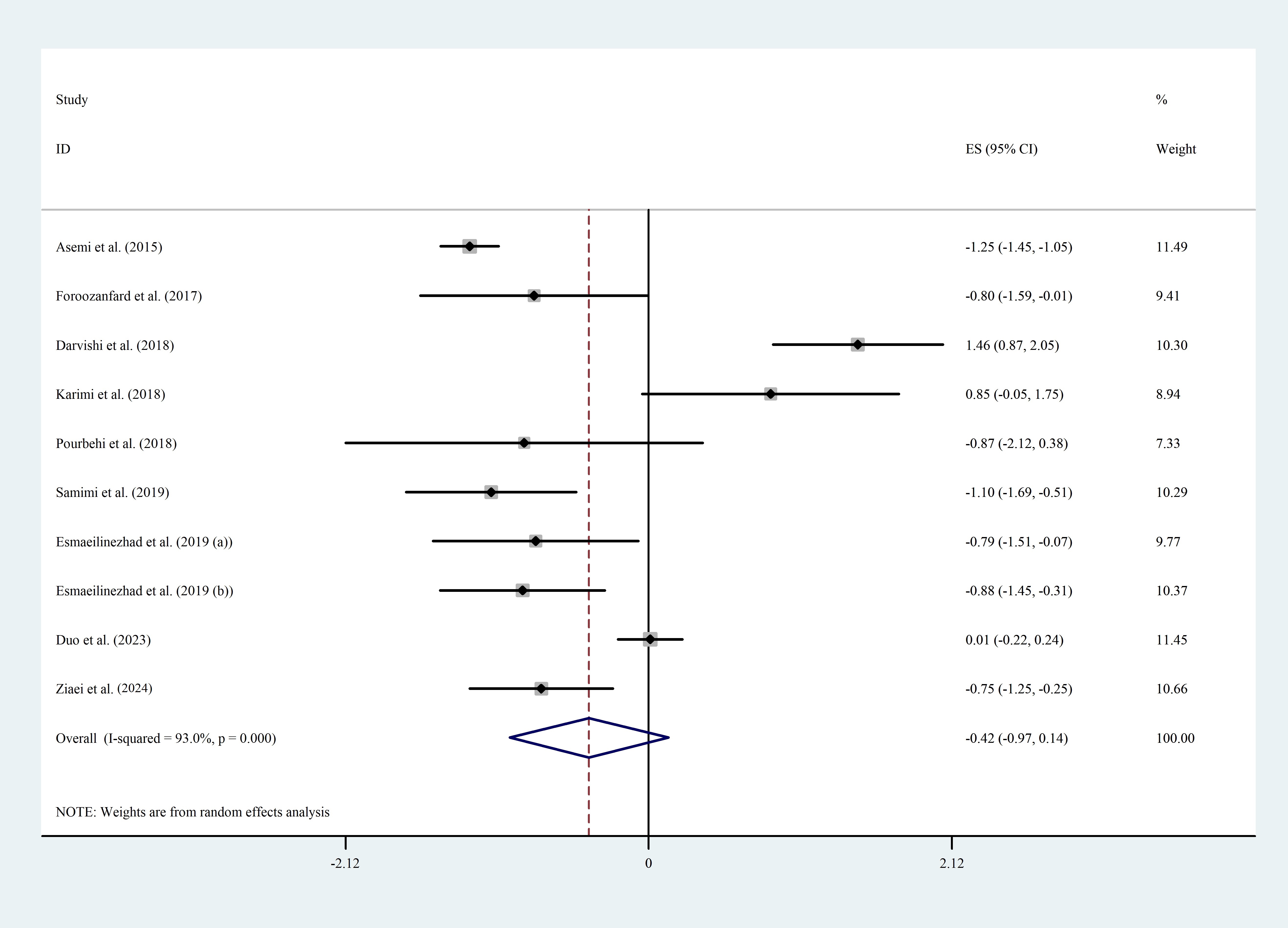

Supplement: Supplementary file 1 [file biomedicines-13-00177-s001.zip › Figure S16_IR .jpg]

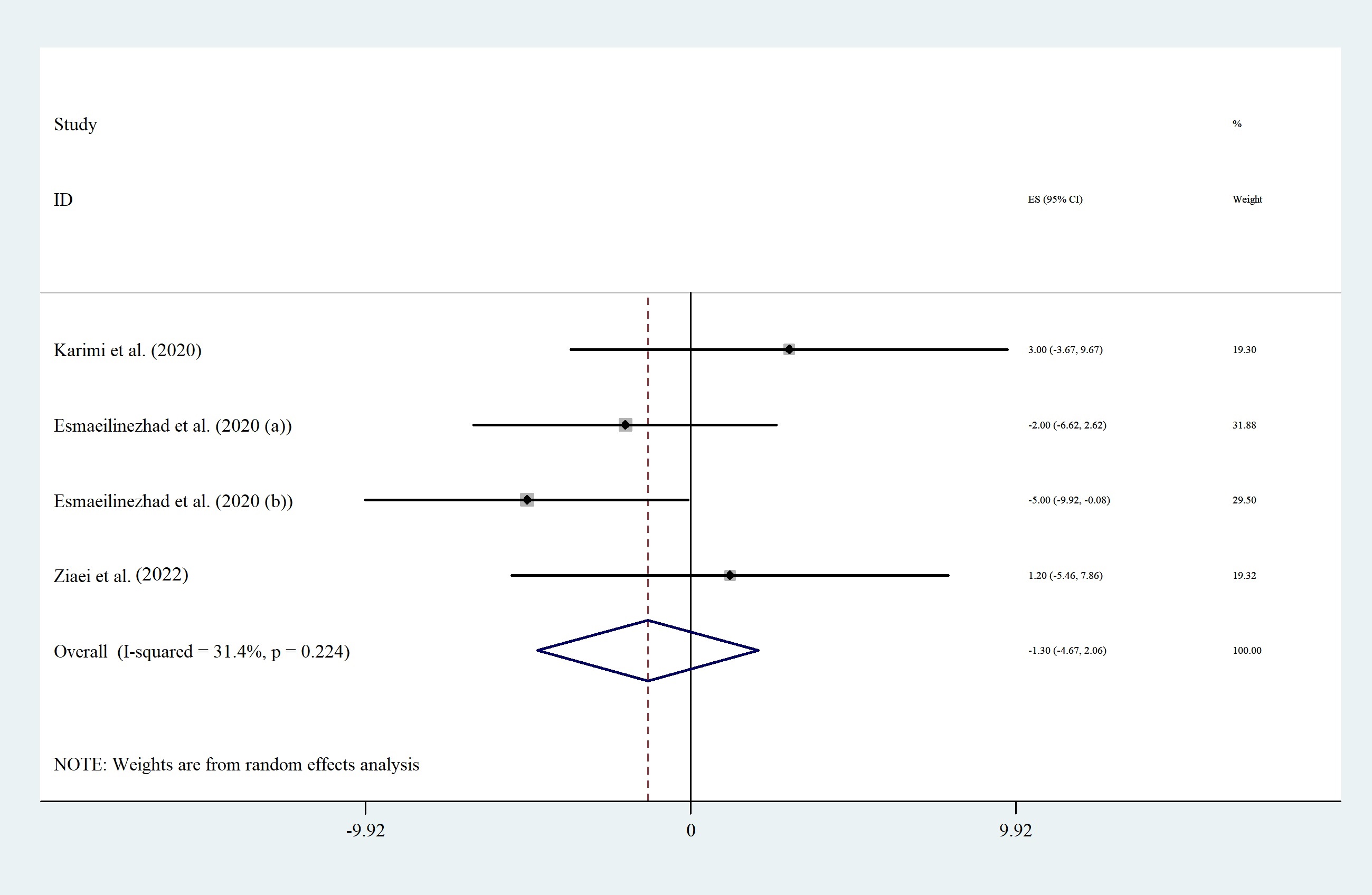

Supplement: Supplementary file 1 [file biomedicines-13-00177-s001.zip › Figure S17_SBP.jpg]

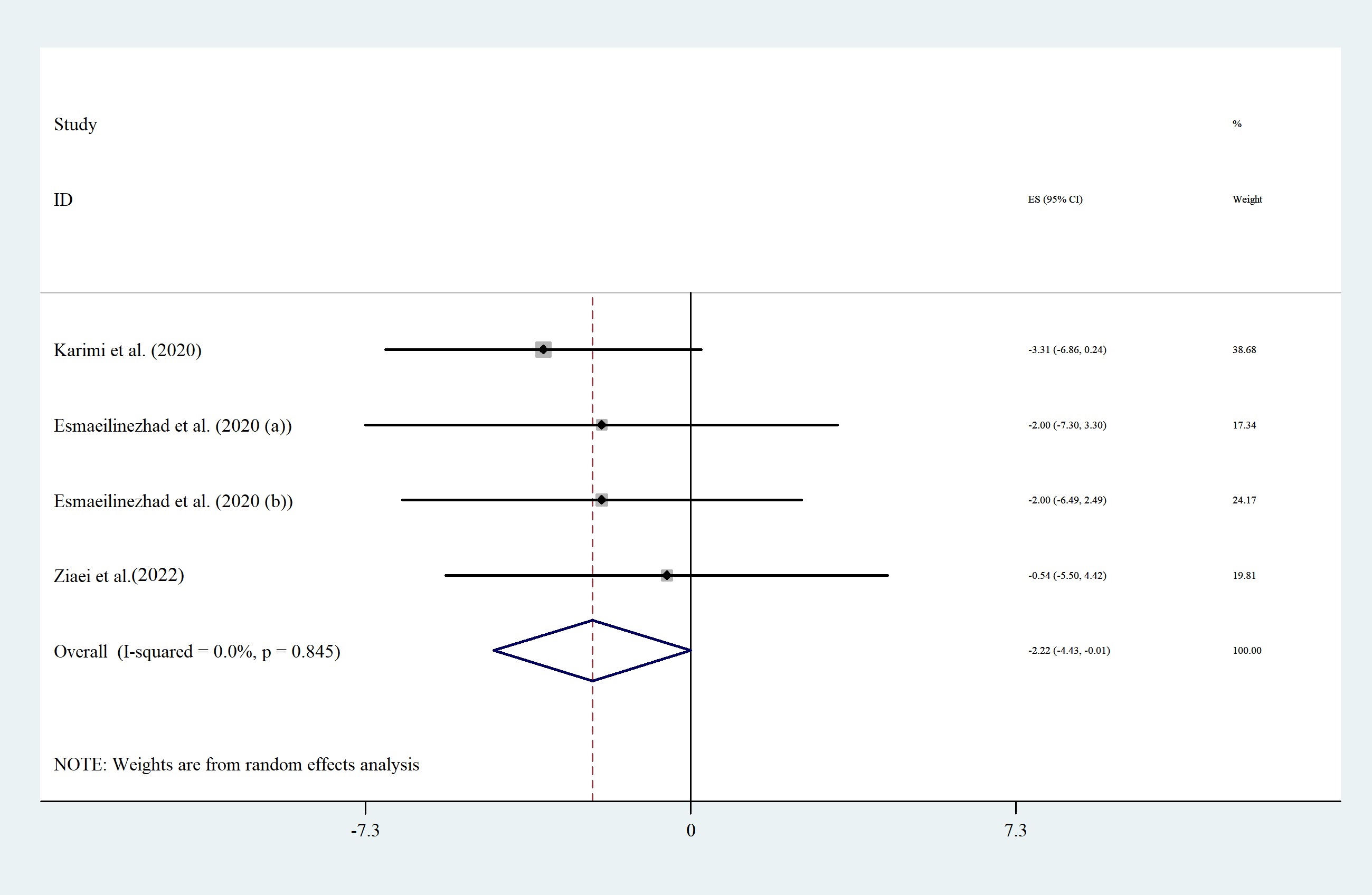

Supplement: Supplementary file 1 [file biomedicines-13-00177-s001.zip › Figure S18_DBP.jpg]

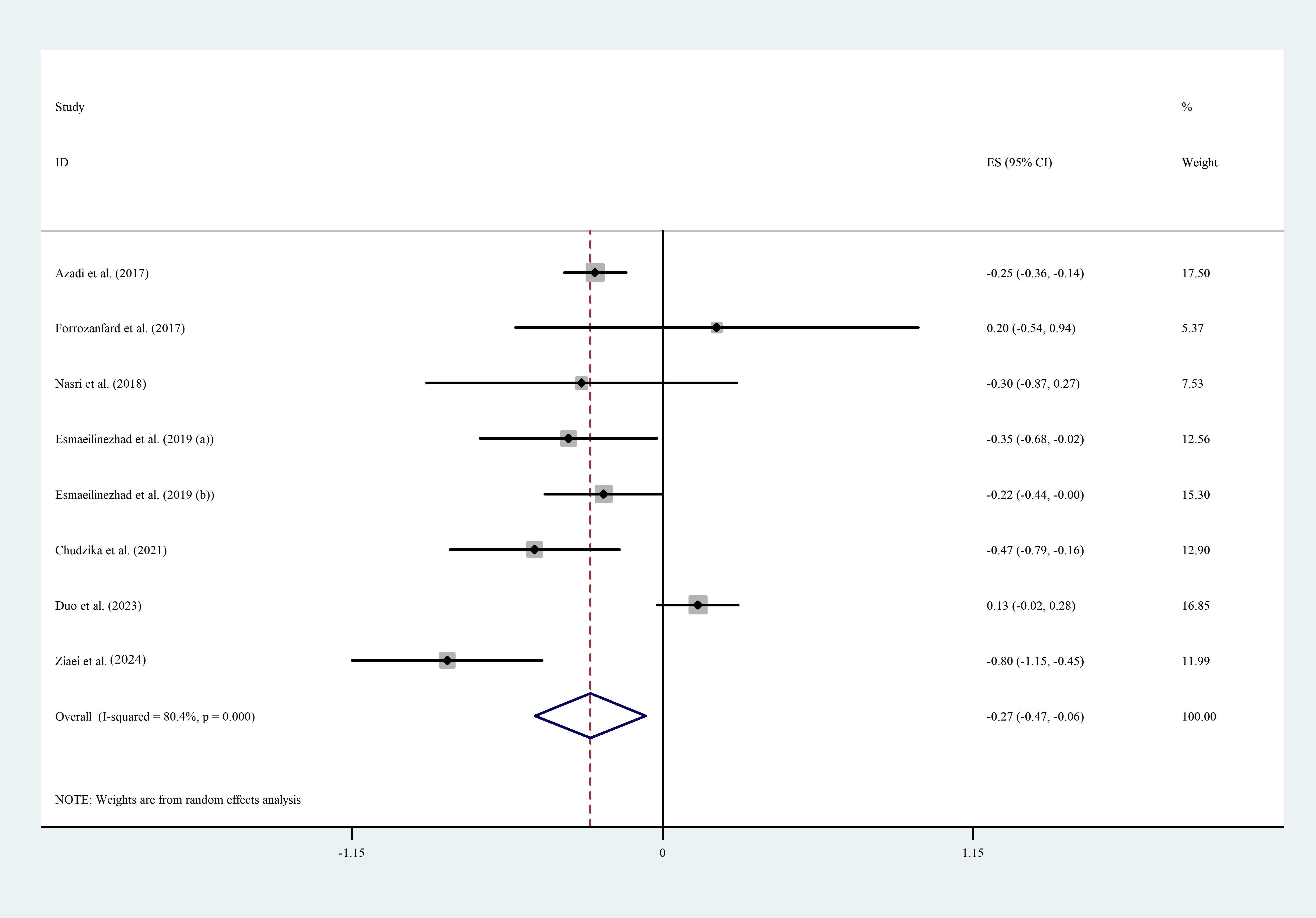

Supplement: Supplementary file 1 [file biomedicines-13-00177-s001.zip › Figure S19_TT .jpg]

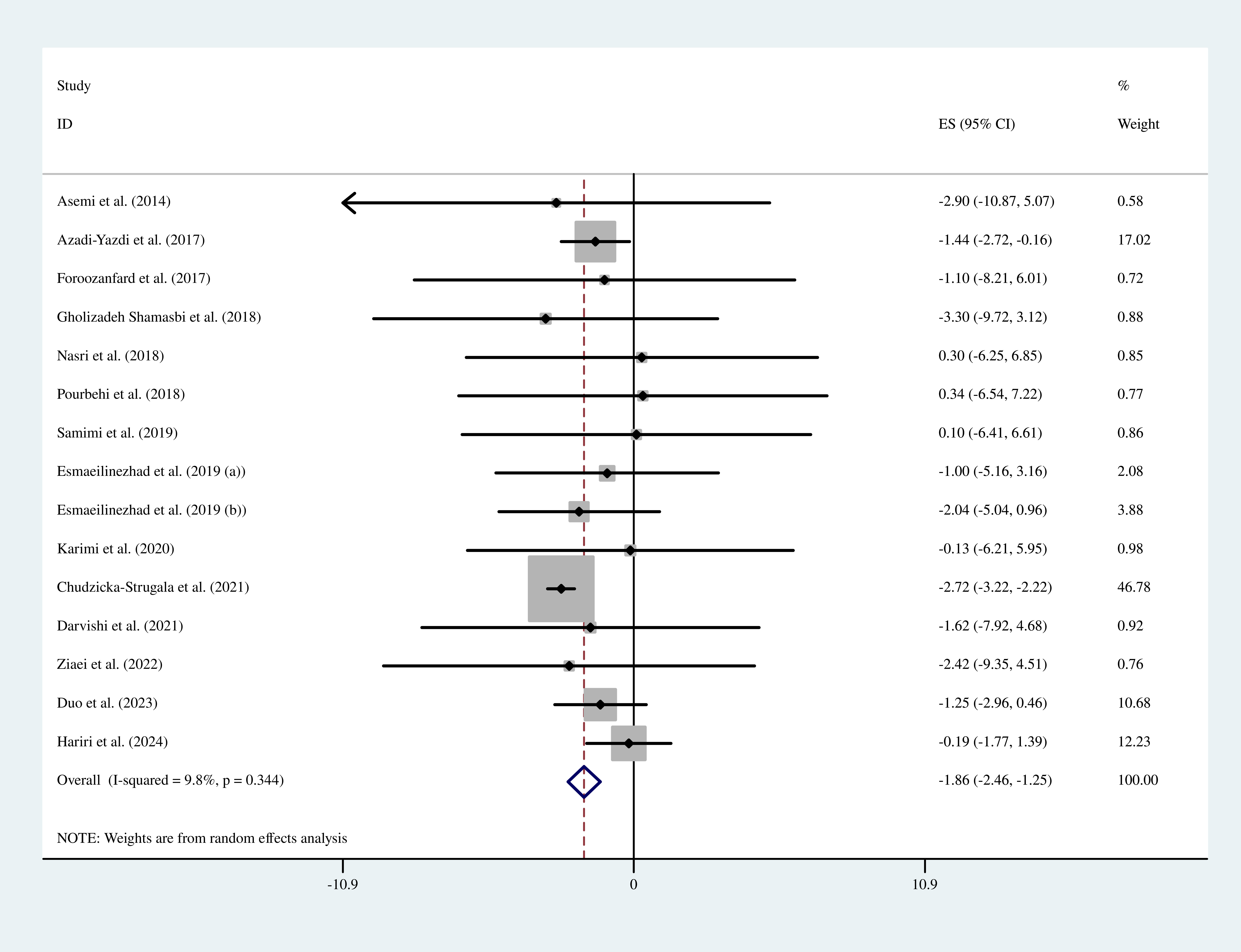

Supplement: Supplementary file 1 [file biomedicines-13-00177-s001.zip › Figure S1_BW_Overall.jpg]

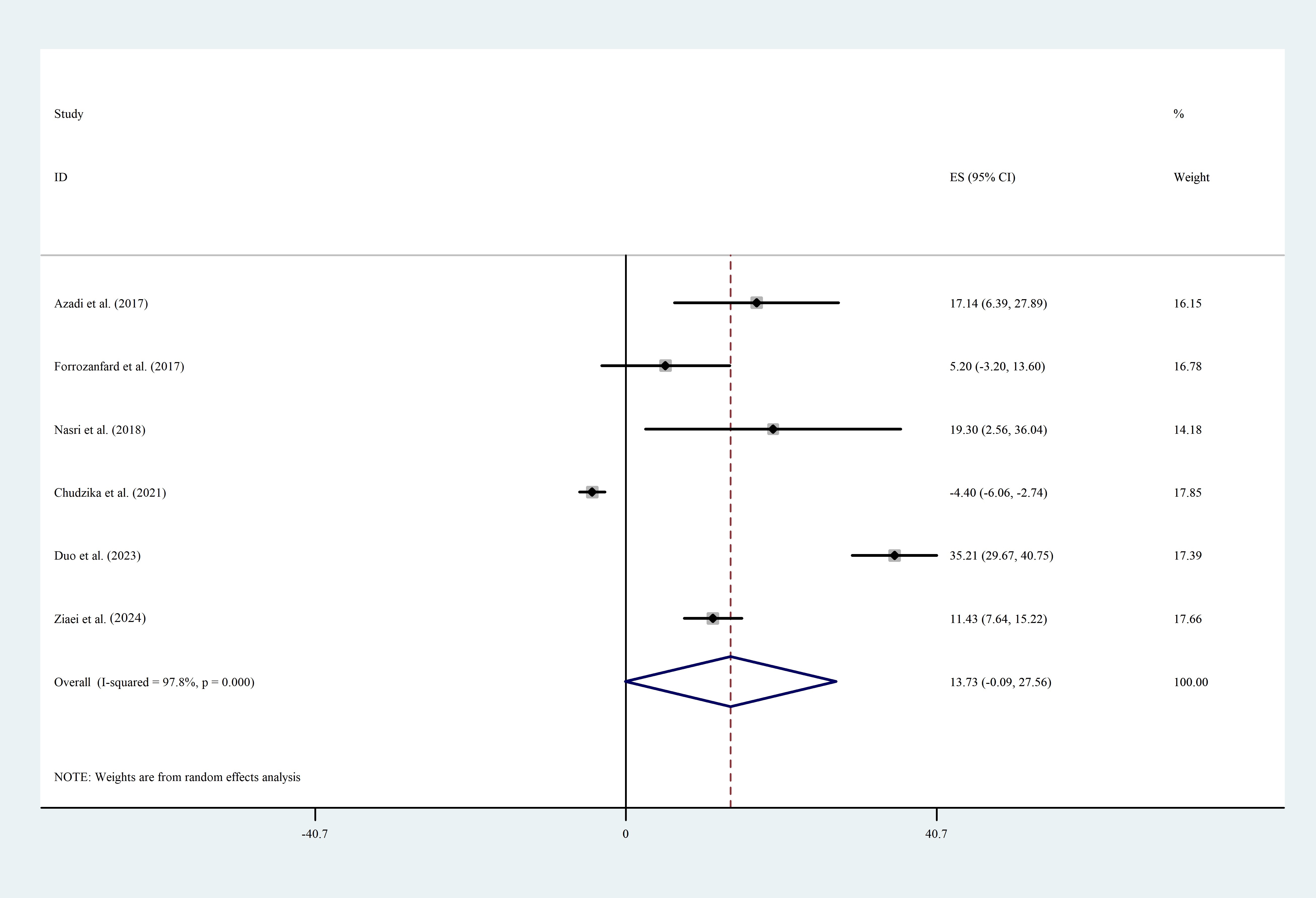

Supplement: Supplementary file 1 [file biomedicines-13-00177-s001.zip › Figure S20_SHBG .jpg]

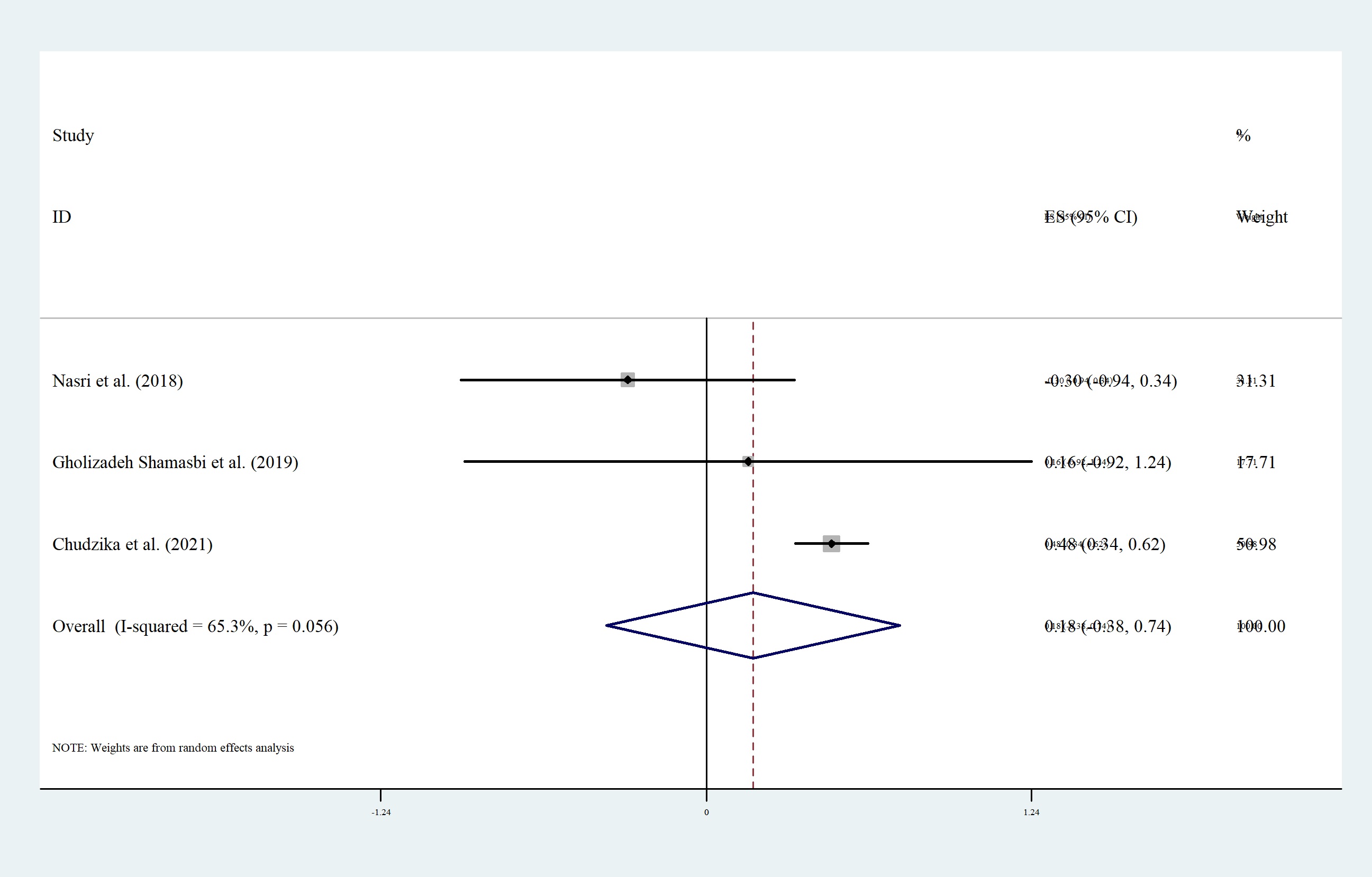

Supplement: Supplementary file 1 [file biomedicines-13-00177-s001.zip › Figure S21_DHEAS.jpg]

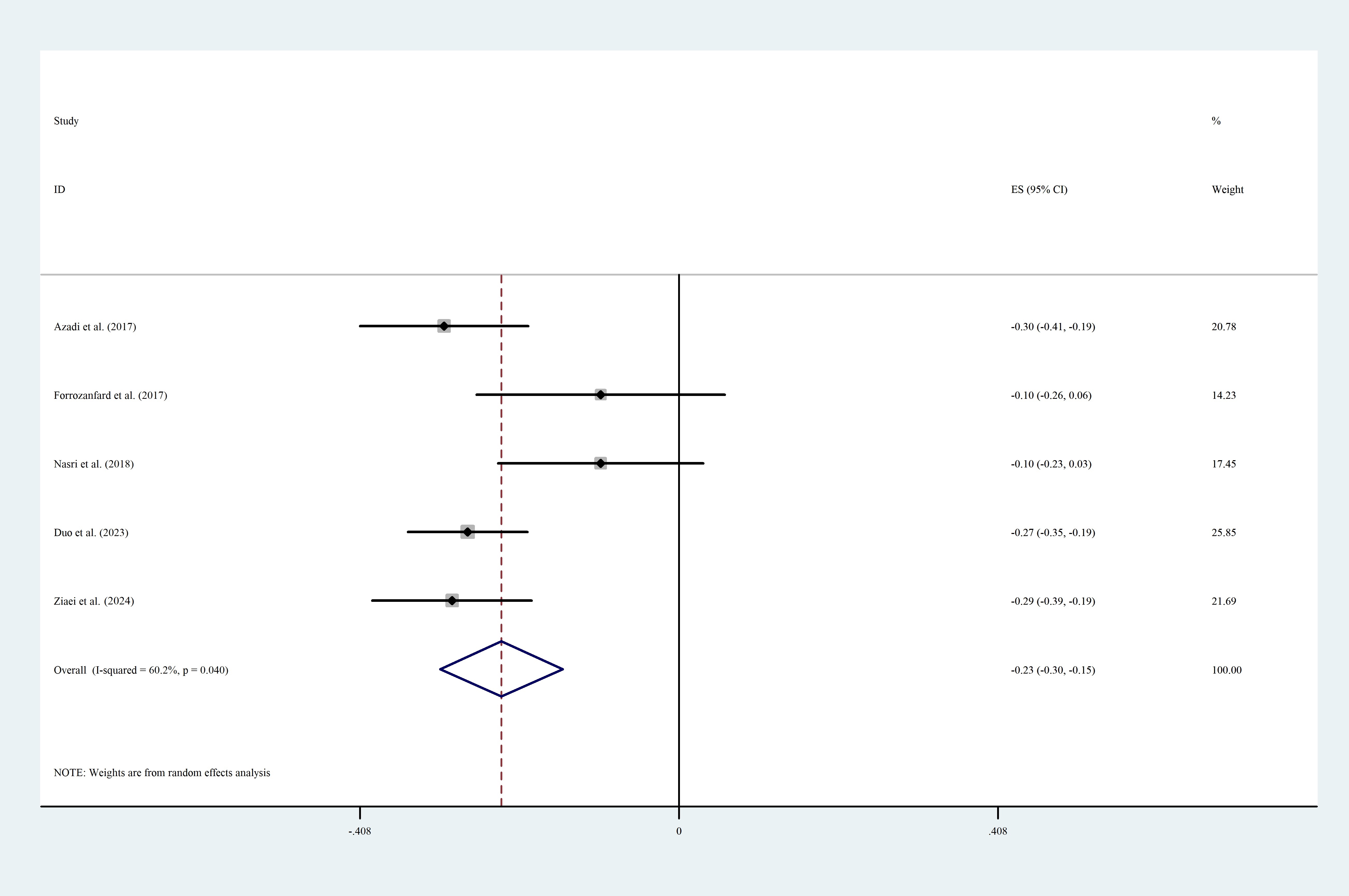

Supplement: Supplementary file 1 [file biomedicines-13-00177-s001.zip › Figure S22_FAI .jpg]

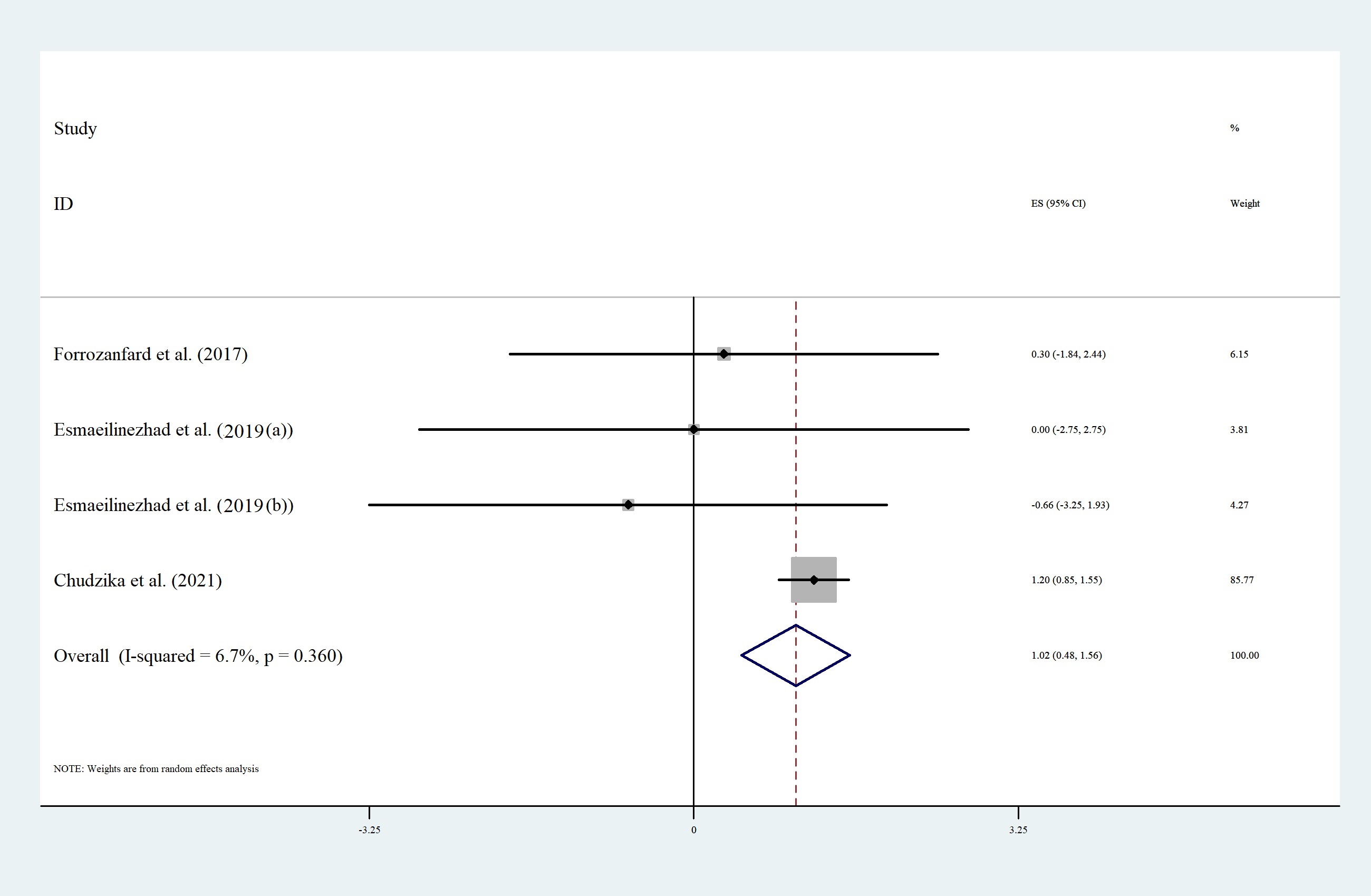

Supplement: Supplementary file 1 [file biomedicines-13-00177-s001.zip › Figure S23_FSH.jpg]

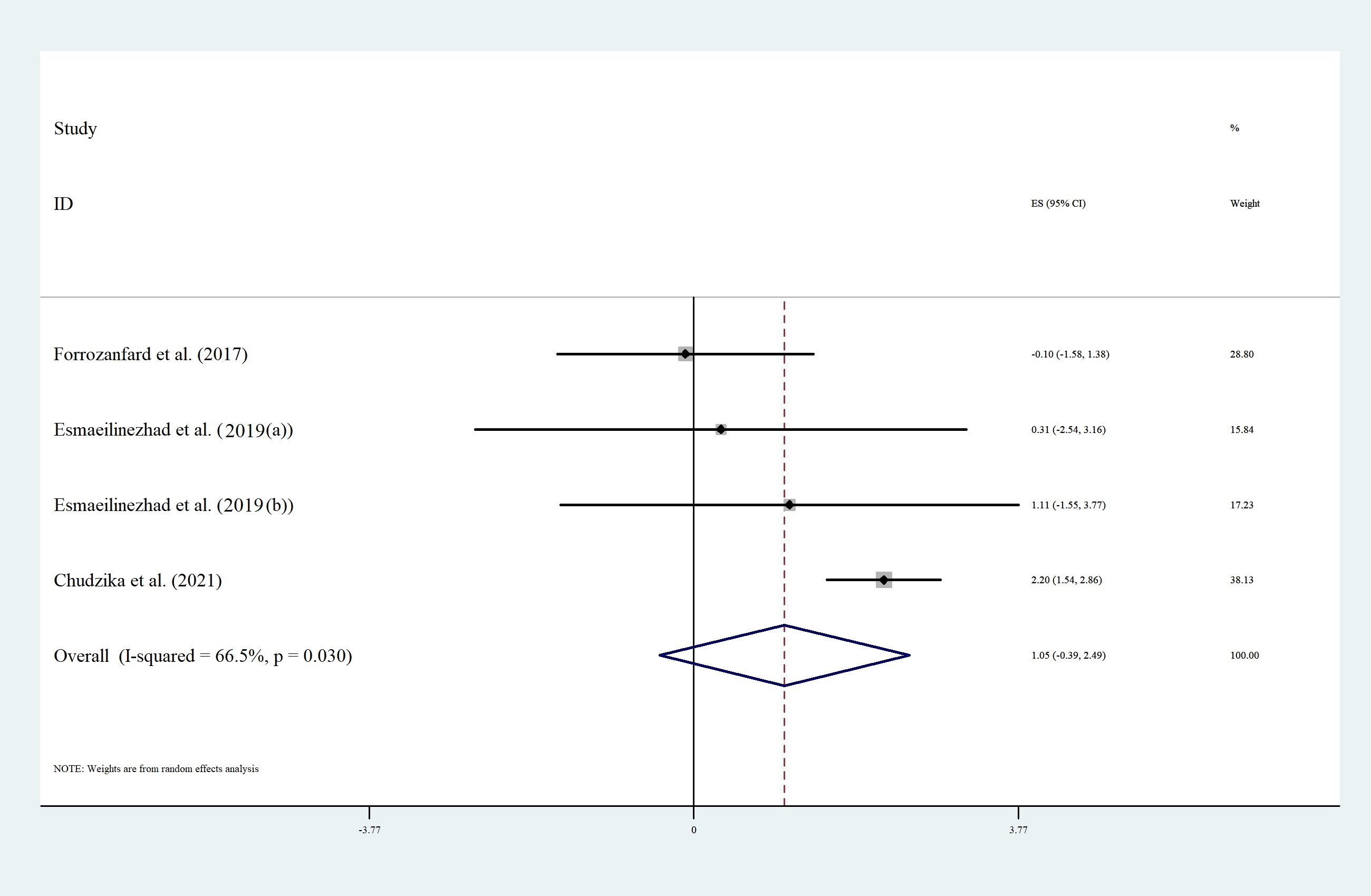

Supplement: Supplementary file 1 [file biomedicines-13-00177-s001.zip › Figure S24_LH.jpg]

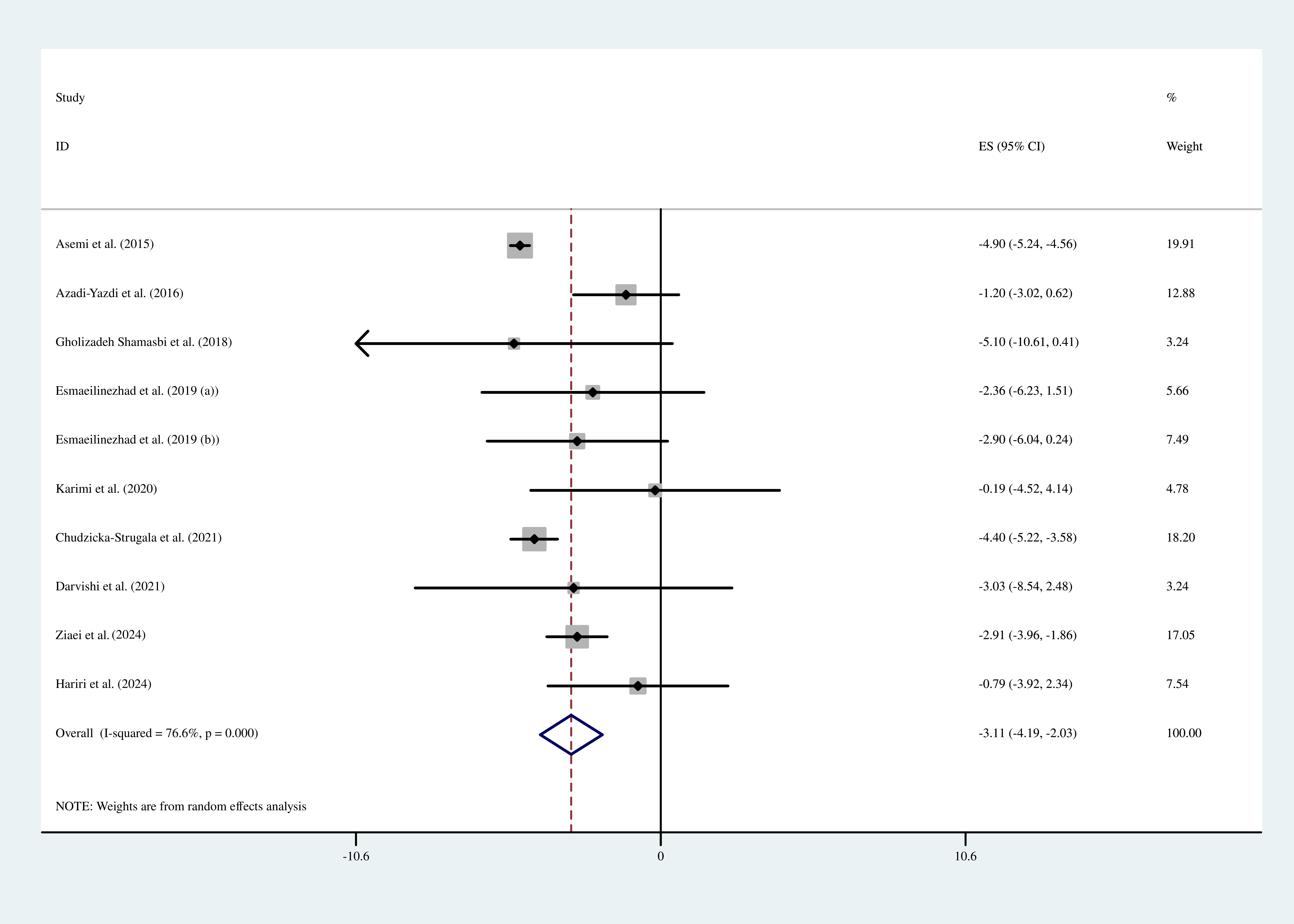

Supplement: Supplementary file 1 [file biomedicines-13-00177-s001.zip › Figure S2_WC_Overall.jpg]

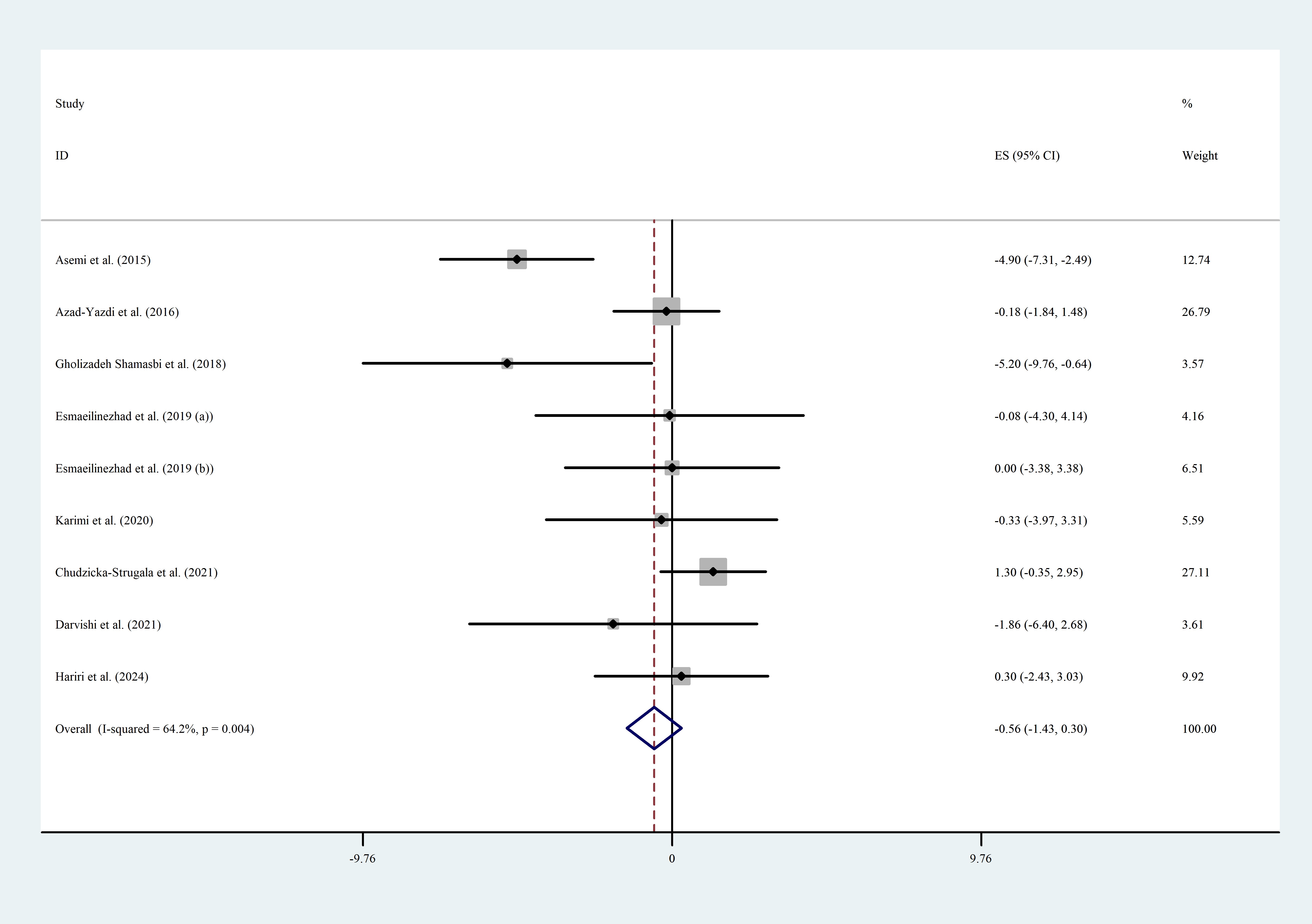

Supplement: Supplementary file 1 [file biomedicines-13-00177-s001.zip › Figure S3_HC .jpg]

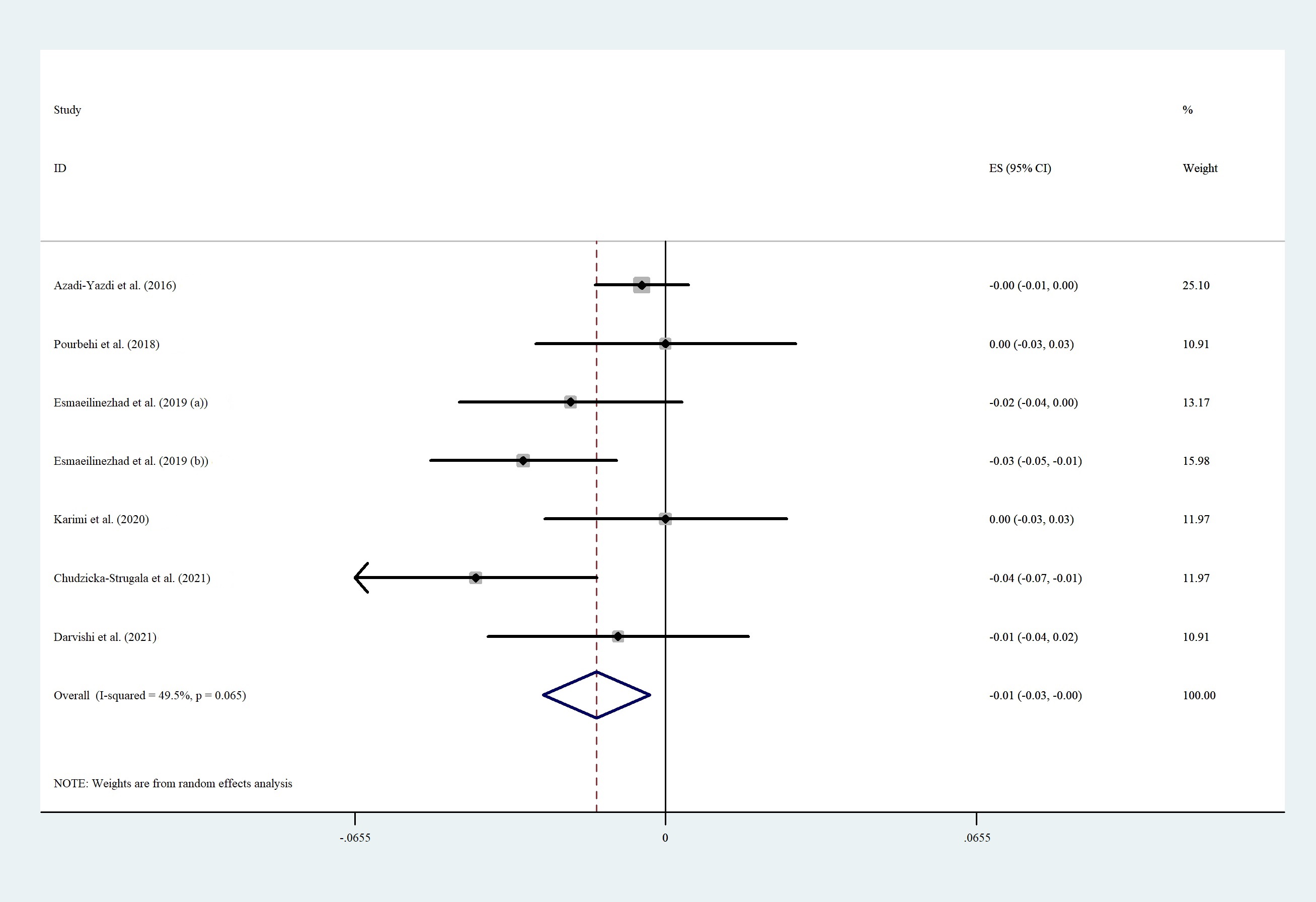

Supplement: Supplementary file 1 [file biomedicines-13-00177-s001.zip › Figure S4_WHR_Overall.jpg]

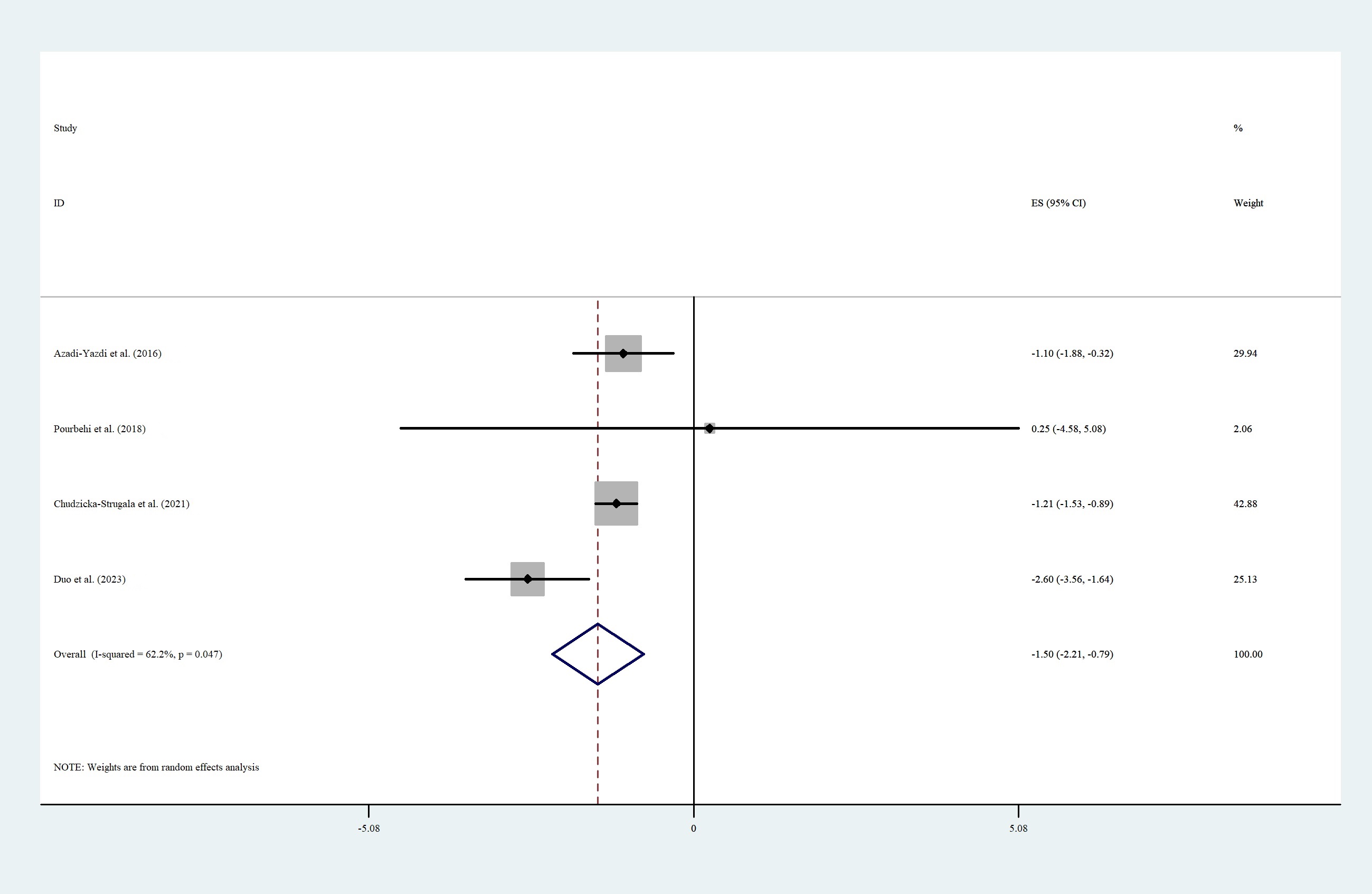

Supplement: Supplementary file 1 [file biomedicines-13-00177-s001.zip › Figure S5_FatMass_Overall.jpg]

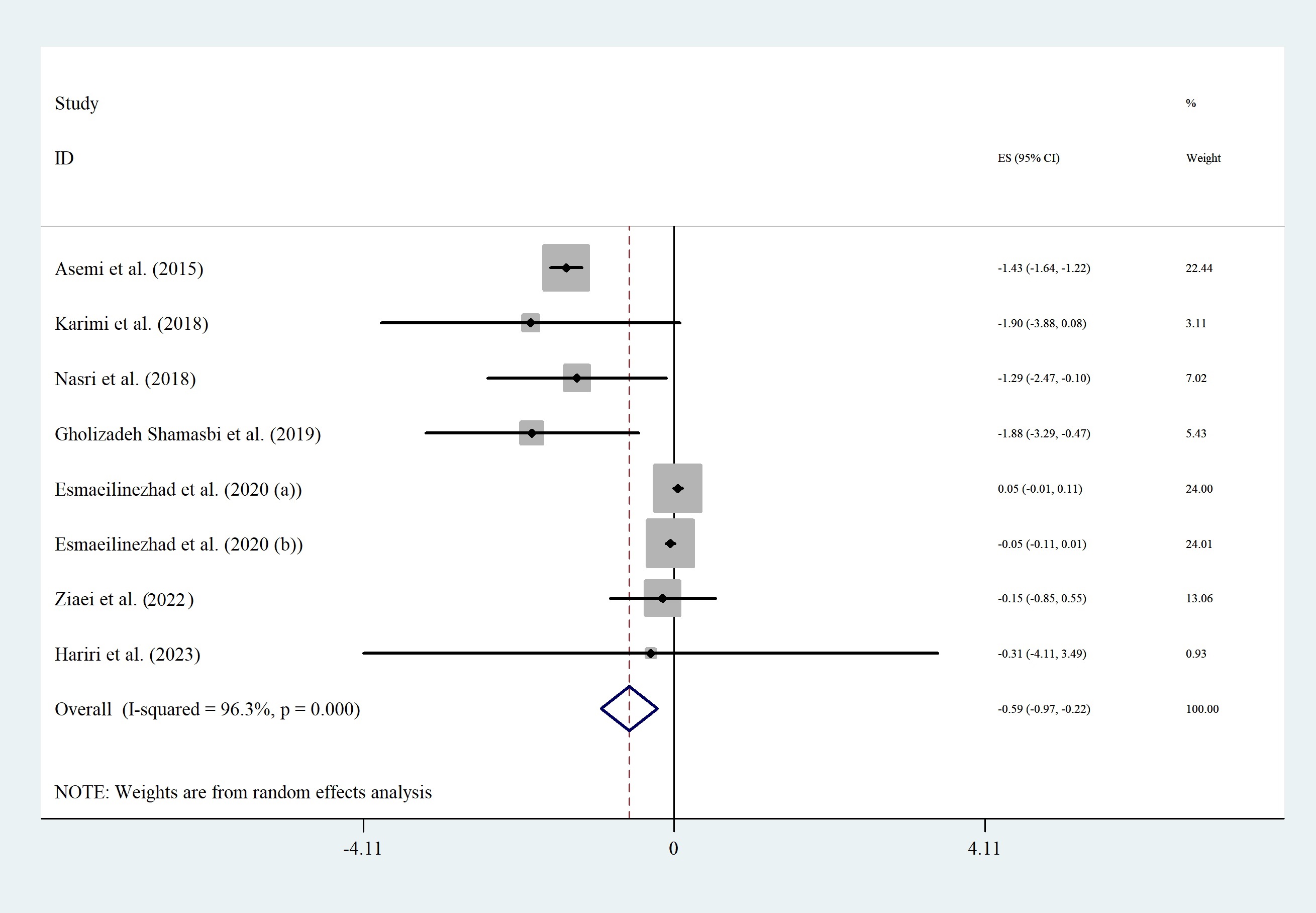

Supplement: Supplementary file 1 [file biomedicines-13-00177-s001.zip › Figure S6_CRP.jpg]

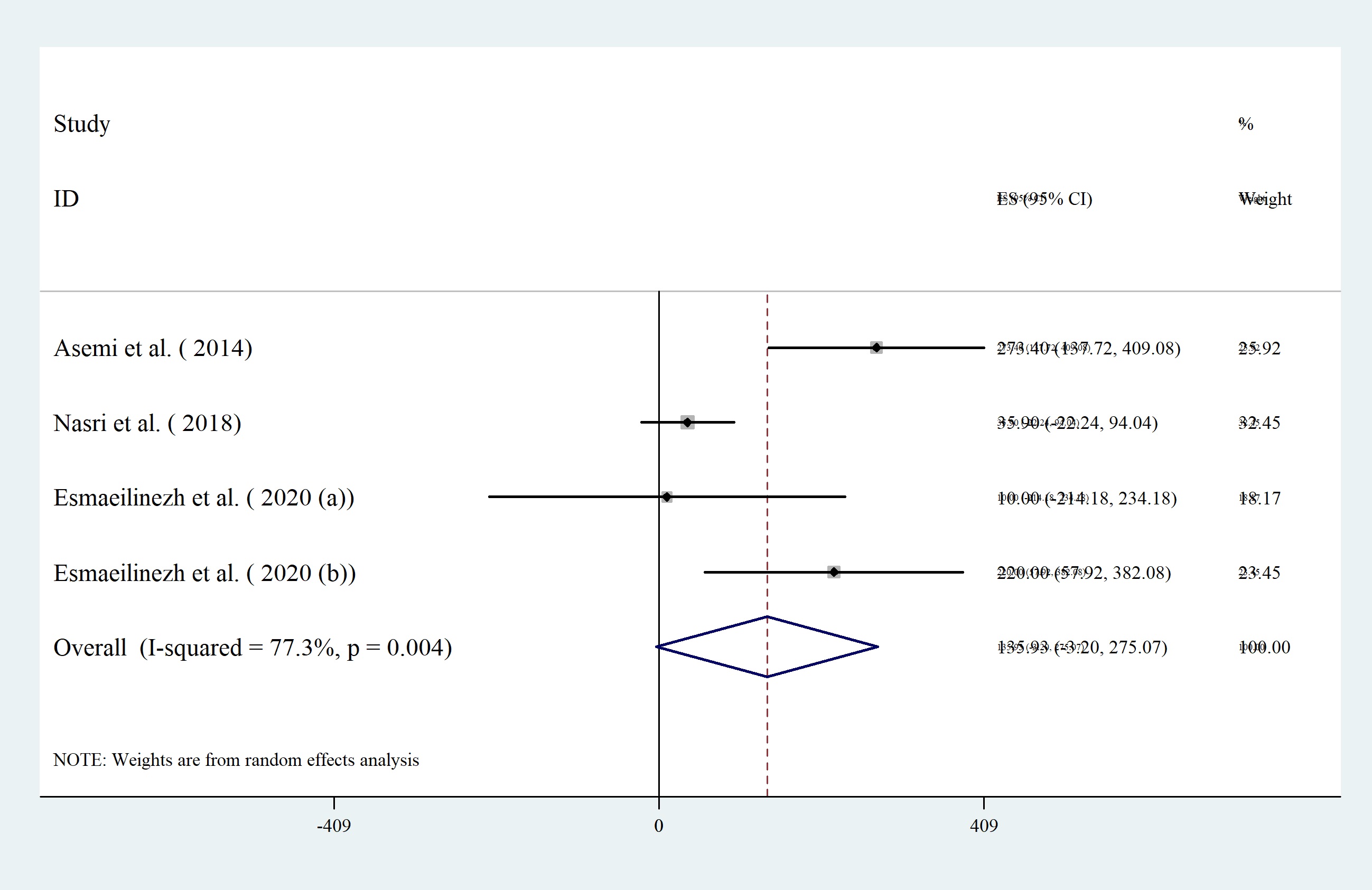

Supplement: Supplementary file 1 [file biomedicines-13-00177-s001.zip › Figure S7_TAC.jpg]

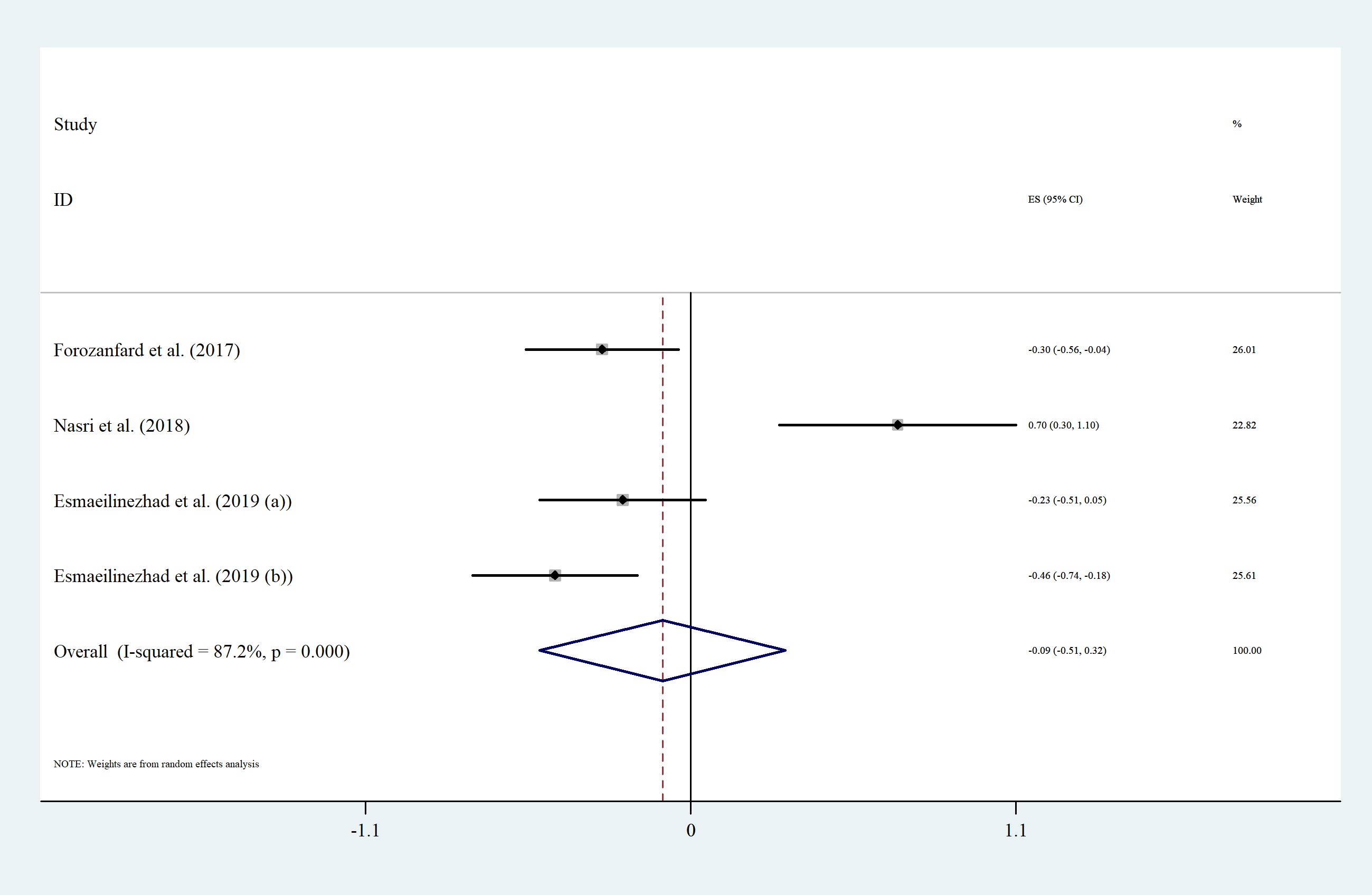

Supplement: Supplementary file 1 [file biomedicines-13-00177-s001.zip › Figure S8_MDA.jpg]

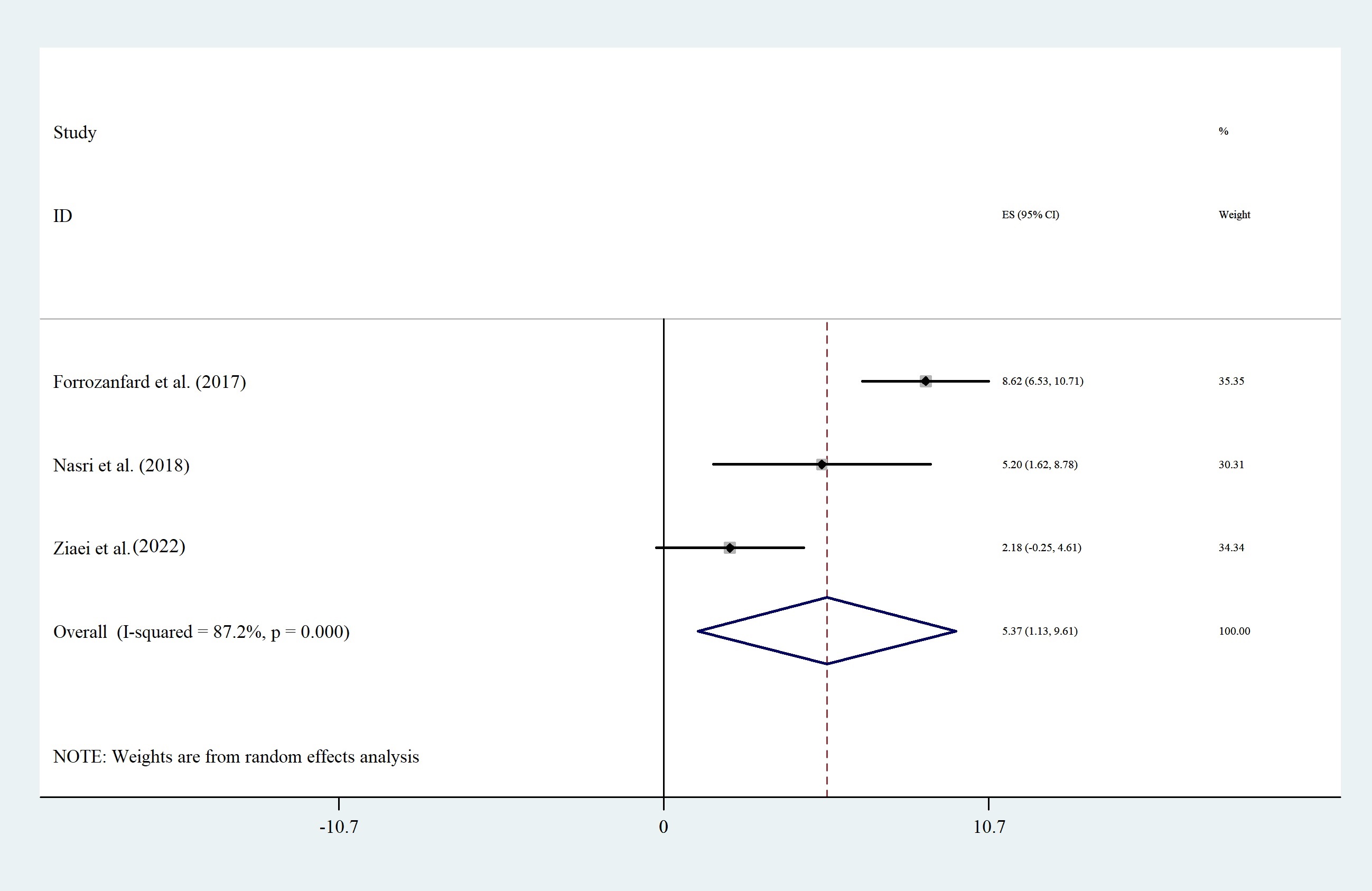

Supplement: Supplementary file 1 [file biomedicines-13-00177-s001.zip › Figure S9_NO.jpg]
